# Using Morphological, Molecular and Climatic Data to Delimitate Yews along the Hindu Kush-Himalaya and Adjacent Regions

**DOI:** 10.1371/journal.pone.0046873

**Published:** 2012-10-08

**Authors:** Ram C. Poudel, Michael Möller, Lian-Ming Gao, Antje Ahrends, Sushim R. Baral, Jie Liu, Philip Thomas, De-Zhu Li

**Affiliations:** 1 Key Laboratory of Biodiversity and Biogeography, Kunming Institute of Botany, Chinese Academy of Sciences, Kunming, China; 2 Plant Germplasm and Genomics Center, Germplasm Bank of Wild Species, Kunming Institute of Botany, Chinese Academy of Sciences, Kunming, China; 3 The Graduate University of the Chinese Academy of Sciences, Beijing, China; 4 Royal Botanic Garden Edinburgh, Edinburgh, Scotland, United Kingdom; 5 National Herbarium and Plant Laboratories, Department of Plant Resources, Godawari, Lalitpur, Nepal; Montreal Botanical Garden, Canada

## Abstract

**Background:**

Despite the availability of several studies to clarify taxonomic problems on the highly threatened yews of the Hindu Kush-Himalaya (HKH) and adjacent regions, the total number of species and their exact distribution ranges remains controversial. We explored the use of comprehensive sets of morphological, molecular and climatic data to clarify taxonomy and distributions of yews in this region.

**Methodology/Principal Findings:**

A total of 743 samples from 46 populations of wild yew and 47 representative herbarium specimens were analyzed. Principle component analyses on 27 morphological characters and 15 bioclimatic variables plus altitude and maximum parsimony analysis on molecular ITS and trnL-F sequences indicated the existence of three distinct species occurring in different ecological (climatic) and altitudinal gradients along the HKH and adjacent regions *Taxus contorta* from eastern Afghanistan to the eastern end of Central Nepal, *T. wallichiana* from the western end of Central Nepal to Northwest China, and the first report of the South China low to mid-elevation species *T. mairei* in Nepal, Bhutan, Northeast India, Myanmar and South Vietnam.

**Conclusion/Significance:**

The detailed sampling and combination of different data sets allowed us to identify three clearly delineated species and their precise distribution ranges in the HKH and adjacent regions, which showed no overlap or no distinct hybrid zone. This might be due to differences in the ecological (climatic) requirements of the species. The analyses further provided the selection of diagnostic morphological characters for the identification of yews occurring in the HKH and adjacent regions. Our work demonstrates that extensive sampling combined with the analysis of diverse data sets can reliably address the taxonomy of morphologically challenging plant taxa.

## Introduction

The Hindu-Kush range and Himalaya regions, generally known as the Hindu Kush-Himalaya (HKH), extends 3500 km from eastern Afghanistan in the West to North Myanmar (Burma) and Southwest China in the East and encompasses two of the global 34 biodiversity hotspots [1]. Yews (*Taxus*) are among the most threatened plants within this region [2]. Excessive harvesting triggered by commercial exploitation for the production of anti-cancer drugs and in some areas intense local use for medicine, timber and fodder, has cleared up to 90% of natural yew populations along the HKH over the last few decades [3], [4]. Despite their great economic importance [5] and domestic values [6], [7], the actual number of species present and their distribution ranges within this region is controversial [8]–[10].

Over the last decade a number of studies have been undertaken in attempts to resolve the taxonomic uncertainties in Asian yews. Some have concentrated on using herbarium specimens to identify suites of morphological characters [11] that could be taxonomically informative, while others have used molecular data to establish phylogenetic and phylogeographic relationships within the genus [12], [13] or a combination of morphological and molecular data to delimitate individual species and approximate their distribution [14]. Despite some progress, uncertainties remain in the number of species present, their precise distributions and ecological preferences, especially in the HKH region. Shah et al. [14] found clear genetic differences between *T. contorta* (as *T. fuana*) and *T. wallichiana* in this region. However, their sampling did not include any from Western Nepal and only a single individual from Central Nepal, this latter area has been proposed as a potential hybrid zone for these yews [11].

Climatic factors are important for the distribution of plants [15], [16], and since even closely related species can have their own unique ecological preferences, climatic modeling may be useful to discriminate between these taxa [17]. Climatic modeling can also help to identify regions of suitable habitat, and as such predict the potential distribution range of species [18], [19].

In the present study, we gathered sets of morphological, molecular and climatic data, predominantly generated from fine-scale collections of yews in the HKH and adjacent regions (NE India, Myanmar, Vietnam and Yunnan, China), particularly from Central Nepal, and applied principal component and maximum parsimony analysis and ecological niche modeling approaches, with the aims to provide an account of the exact number of *Taxus* species and their precise distribution boundaries along the study area, with special focus on the proposed hybrid zone in Central Nepal. This study is of high significance for the conservation and sustainable management of the yew resources in this region.

## Materials and Methods

### Taxon Sampling

**Figure 1:**
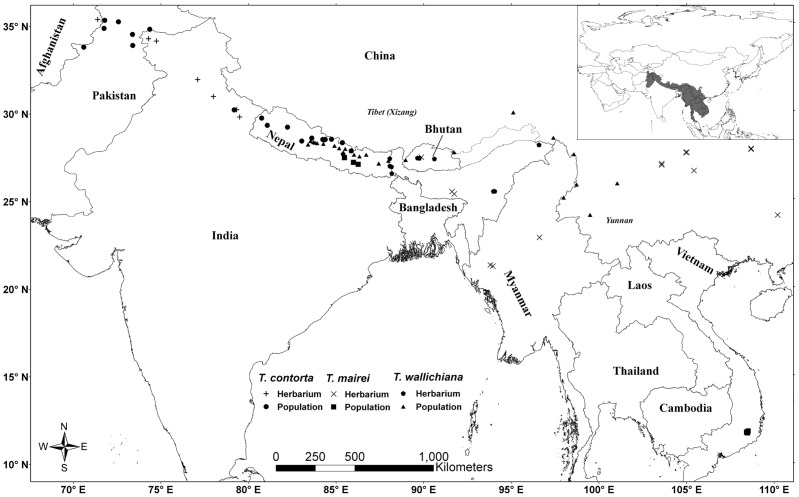
Sampling localities. Map of the Hindu Kush-Himalaya and adjacent regions, showing localities of the sampled populations and herbarium specimens of *Taxus* included in the present study. A total of 743 population level samples and 47 herbarium specimens were used to delimitate *Taxus* along the HKH and adjacent regions.

Population level samples (4–20 individuals per population) were collected from 2003 to 2011 throughout the entire HKH and adjacent regions, and a total of 743 individuals from 46 populations were sampled (Figure 1). Voucher specimens for each individual sample were collected and deposited in Kunming Institute of Botany, Chinese Academy of Sciences Herbarium (KUN), National Herbarium and Plant Laboratories (KATH) and Royal Botanic Garden Edinburgh Herbarium (E) (Table S1). The highest density of collections came from Central Nepal, a putative hybrid zone between the western and eastern Himalayan yew species that has been identified in previous studies [10], [11]. For areas from where we could not collect samples (NW India: Jammu & Kashmir, Himachal Pradesh; NE India; Bhutan; Myanmar; Vietnam), a total of 36 herbarium specimens, including two type specimens, deposited in the herbaria E and Royal Botanic Gardens Kew Herbarium (K) were consulted to fill the gap in sampling. During our field trips to Nepal and herbarium examinations, we noticed that some specimens morphologically resembled *T. mairei* which mainly occurs in South China. Therefore, eleven samples from the center of distribution of *T. mairei* (Guizhou, Guangxi, Fujian, Zhejiang) [10], [11], [13], including the type, were included in our analysis to confirm the presence of *T. mairei* along the HKH and adjacent regions. The morphological dataset thus included 790 samples in total.

For molecular work we sequenced up to twenty individuals for chloroplast DNA (cpDNA) *trn*L-F and five individuals for nuclear ribosomal DNA (nrDNA) ITS from each of the 46 field collected populations. To geographically complement our field-collected material, three individual samples of *T. mairei* (1 from Vietnam, 2 from China) and five herbarium samples (*T. contorta*, India; *T. mairei*, Bhutan, India, Myanmar; *T. wallichiana*, Bhutan) were included. The majority of the sequences showed little or no sequence variation within species (data not shown) thus we used a selected number of sequences to represent genetic diversity and the species distribution ranges. Thus, in the final molecular analysis 36 samples were included. Only 33 ITS sequences were incorporated in the phylogenetic analysis, since sequencing failed for the Bhutanese sample BT5, and two hybrid samples showing multiple polymorphic bases were excluded (see Results and Table S4).

The field collected populations and herbarium specimens were tentatively identified according to the work of Möller et al. [11] and Shah et al. [14]. Details on sample locations, corresponding vouchers and GenBank accession numbers of the samples used in the molecular analysis are provided in Table S1.

### Measurements and Morphometric Analysis

The morphological matrix included 27 characters (1 bud and 26 leaf characters) used in Möller et al. [11], with some modifications for character 11 which we redefined as ‘leaf arrangement’ and by adding an extra character state (2 = irregularly pectinate) (Table S2). Möller et al. [11] defined this character based on the insertion of the leaf on the stem, while Shah et al. [14] considered the leaf arrangement on the branchlets due to the twist of the leaf base. Here we used the latter approach which is more reliable to score. Though Shah et al. [14] recorded *T. contorta* (as *T. fuana*) with mostly spiral leaf arrangement, in our larger sampling sets, we found that this description was not accurate for this species. It implies a regularly omnilateral leaf position (as in *T. cuspidata*) but in *T. contorta* the leaf arrangement is intermediate between pectinate and spiral. Otherwise, the measurement procedures followed Möller et al. [11]. Six continuous characters were found not to be normally distributed and the data were log transformed, before all data were subjected to a principle component analysis (PCA) and the results plotted in two dimensional PCA scatter plots.

Following the Kaiser criterion [20], the first three PCA coordinates were selected and were evaluated further by a discriminant analysis to test the statistical significance of the groupings observed in the PCA scatter plot. A one-way analysis of variance (ANOVA) with *Post hoc* test (Tukey's HSD) was performed to determine whether the means of the continuous characters of the different PCA groups were significantly different from each other. For the same purpose a Chi-square test was performed for the discrete characters. All analyses were performed in SPSS 16 (SPSS Inc., Chicago IL, USA) and Minitab 15 (State College, PA: Minitab, Inc., USA).

### DNA Extraction, PCR, Sequencing and Phylogenetic Analyses

DNA was extracted from silica-gel dried leaf materials of all field-collected individuals and from leaves of selected herbarium specimens. DNA extraction, PCR amplification and sequencing of nrDNA ITS and cpDNA *trn*L-F followed Shah et al. [14]. Sequences were edited and assembled using Sequencher 5.0 (Gene Codes Corp., Ann Arbor, MI, USA) and aligned with ClustalW in BioEdit followed by minor manual adjustments [21]. Indels in the *trn*L-F matrix were coded according to the simple gap coding method [22]. Maximum parsimony (MP) analysis was performed in PAUP 4.0b10 [23], using the heuristic search option with 1000 random additions and TBR swapping and MulTrees on. Branch support was provided by a bootstrap analysis of 10000 replicates of heuristic searches, with MulTrees on and TBR swapping off. Consistency indices (CI) and retention indices (RI) were obtained in PAUP.

### Species Occurrence and Climate Data

**Figure 2:**
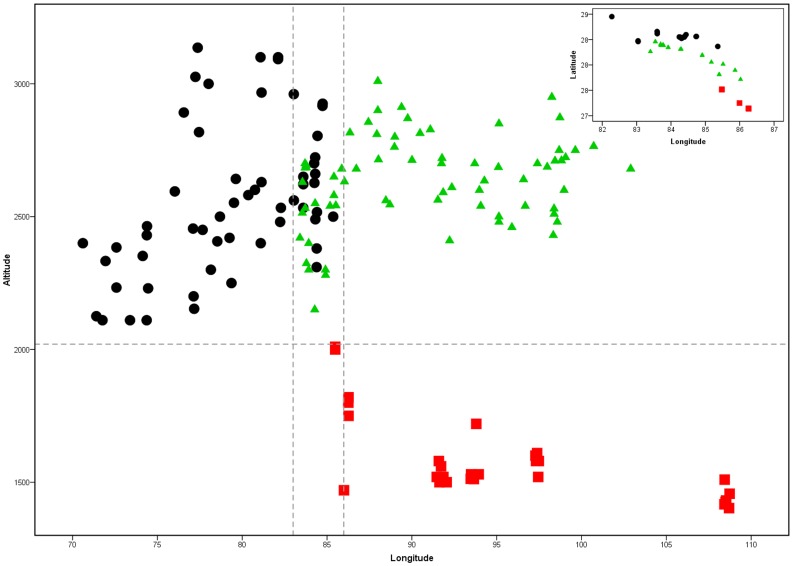
Yew distributions. Distribution of yews in the Hindu Kush-Himalaya and adjacent regions with longitude and elevation. Top right inset shows non overlapping parallel geographic distribution of the three species of *Taxus* in an area between 84°00′–86°30′ longitude.

Yews in the HKH region are reported from eastern Afghanistan to Yunnan in Southwest China. They occur along the foothills of the Himalayas in a narrow band in central Nepal and the small area of SW Xizang (Jilong) in China between longitudes 83° 00′ - 86°30′ (Figure 2, inset). The HKH region is geographically highly complex. It comprises more than ten peaks above 8000 meters and innumerable peaks below 7000 meters [24]. There is thus likely extensive small-scale climatic variation. At present, the highest resolution climate data that cover the entire HKH region are the WorldClim grids (http://www.worldclim.org), which were created by interpolating climatic data from weather stations around the world using a thin plate smoothing spline, at a resolution of 30 arc-seconds (∼1 km^2^ per pixel) [25].

We obtained data for 19 bioclimatic variables (at 30 arc-second resolution) from WorldClim, and used expert pre-selection to constrain the candidate environmental predictors to a set of variable that are likely to be eco-physiologically important and uncorrelated (|Pearson’s R| ≤0.7). Although MaxEnt has an inbuilt regulization mechanism, selecting proximal variables out of sets of correlated variables is important when the models are projected to other areas [26]. A table showing the correlations between all the environmental variables and which variables were included into the model can be found in Table S3.

Previous studies in DNA barcoding and population genetics have suggested that the three *Taxus* species analyzed here are not sister species [13], [27], [28]. Therefore we did not trace their evolution and the ecological mode of speciation [29]. Instead, the focus of this study is to develop a simple climatic niche model and evaluate the climatic features of each yew species.

### Ecological Niche Modeling

We developed niche models using MaxEnt [26], [30], – a machine learning algorithm for ecological modeling from presence-only records, which has consistently performed well in cross-model comparisons [31]. MaxEnt estimates the probability of occurrence based on the density of the covariates at the presence sites, and in their density in the entire study area (background data). It searches for the solution that has maximum entropy (i.e. is closest to a null model whereby a species/species group has no environmental preferences), subject to the constraint that the means of the environmental covariates are close the means across the presence locations [32]. We developed the models at a 1 km resolution using the default settings for MaxEnt version 3.3.3k (allowing for transformations of the covariates with the default thresholds for conversion, removing duplicate presence records, maximum number of background points = 10000; maximum number of iterations = 500; convergence threshold = 0.00001; fit regulization parameter = 1; default prevalence = 0.5). To evaluate model performance we ran each model with 10-fold cross-validation.

The model input data (minus duplicates) consisted of 56, 67 and 28 presence records for *T. contorta*, *T. wallichiana* and *T. mairei* respectively. From previous studies [11], [13], [14], [28], [33] and our extensive field trips along the Himalayas we are confident that these records accurately capture the distribution extent of yew species in this region. It has become increasingly clear that extent of the area used for model development can have strong effects on model prediction, validation and model comparison and therefore needs to be delineated with upmost care to only encompass the area that is within the dispersal capability of the species in question, and to represent this area entirely [34], [35]. Unfortunately, very little is known about the dispersal of our focal species. We therefore produced the minimum convex hull that encompasses all distribution records for a given species plus a one degree (c. 110 km) buffer to demarcate the modeling area for each species.

### Comparison of Ecological Niches

Comparison of ecological niche models can serve to compare species environmental requirements. However, for these comparisons to be biologically meaningful they need to account for the differences in the environments available to the species [34], [36]–[39]. Broennimann et al. [36] showed that, if the frequencies of different environments that occur across the ranges of the two species are not accounted for, niche overlap is systematically underestimated. Put simply, the absence of species A in an environment that is occupied by species B but that is out with the dispersal capability of species A is insufficient to conclude that therefore species A and species B have different environmental requirements.

Here we used two methods to explore whether the species have similar environmental requirements. First, we followed a framework and scripts developed by Broennimann et al. [36] that allow for statistical tests of niche hypotheses, whilst correcting for any bias associated with the availability of environments for the species. Some pre-selection of environmental variables was carried out to exclude variables that are ecologically irrelevant. We tested each of the species pair combinations separately. The procedure involves three steps [36]: 1) a multivariate analysis of the environmental space is performed, the environmental space is gridded in *r*×*r* cells of unique environments, and the density of occurrence of the species and of the different environments within this environmental space are calculated. The species occurrences are smoothed using a kernel density method to account for sampling gaps and biases, and to ensure that the results are independent of the grid resolution *r*. 2) the amount of niche overlap between the species within this environmental space is calculated, and 3) statistical tests for niche identity and niche similarity (*cf*. Warren et al. [40]) are performed. Regarding the method for the initial multivariate analysis of environmental space, we used a two-dimensional PCA, calibrated on the entire environmental space available to the two species in question (“PCA-env”). This approach has been identified as most robust to errors and biases associated with the estimation of niche overlap by Broennimann et al. [36]. For the resolution *r* of the environmental space and the smoothing parameters of the kernel density function were used the defaults set by Broennimann et al. [36] (*r* = 100; Gaussian kernel with a smoothing bandwidth that defaults to 0.9 times the minimum of the standard deviation and the inter-quartile range divided by 1.34).

Second, we tested the performance of models developed for each species to predict presences of the other two species [39]. The underlying assumption here is that if the species have different ecological requirements, models developed for one species should be bad predictors for the presence of another species. Model performance was measured using the area under the receiver operating characteristic curve (AUC). The predictions included into the AUC score were restricted to areas that are inside the range of the environmental variables in the training data (areas that scored ≥0 on a multivariate similarity surface [41] implemented in MaxEnt). This restriction was imposed to avoid any biases when comparing the model performance that arise due to models being less likely to be accurate when they are extrapolated outside the environmental space sampled by the training. A problem associated with this approach is that models may generally perform less well when they are predicted to a new region [39]. Following Godsoe et al. [39] we therefore undertook an additional test whereby we developed all models excluding the contact zone (Nepal) and then predicted models to that area, as such forcing each model to predict to a new area.

## Results

### Morphometric Analysis

**Figure 3:**
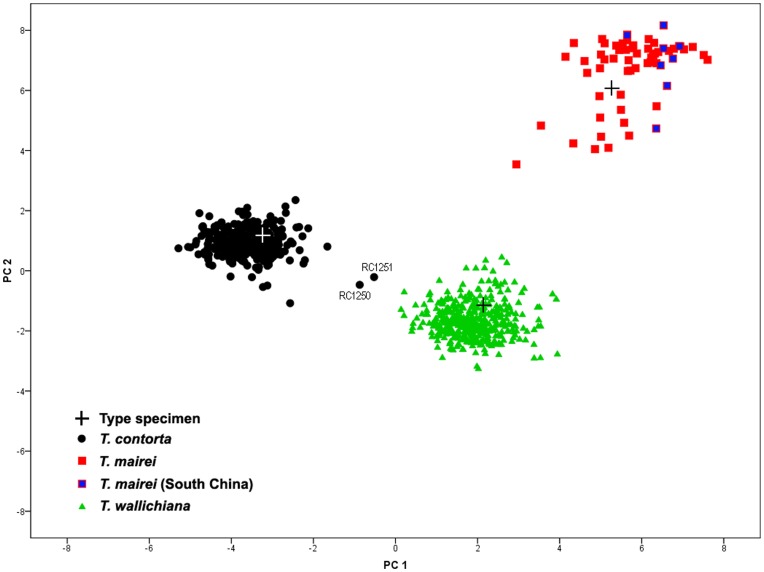
Principal component analysis (PCA) of morphological characters. PCA scatter plot of the first two coordinates based on 27 morphological characters for 743 population level samples and 47 herbarium specimens of *Taxus* from the Hindu Kush-Himalaya and adjacent regions.

The first three components of the morphological PCA explained 37.5%, 20.9% and 7.5% variation respectively, giving a combined variance explained of 65.9% (Table 1). The scree plot [42] and Kaiser criterion [20] suggested that the first two axes were sufficient to be displayed, representing 58.4% variation. The PCA scatter plot showed three clearly distinct groups of specimens, corresponding to *Taxus contorta*, *T. wallichiana* and *T. mairei*, with the type specimens falling in the center of each respective species cluster (Figure 3). The species clusters did not overlap and were well separated, with the first axis separating all three groups, while the second axis separated the *T. mairei* samples from those of the other two species. Those belonging to *T. contorta* and *T. wallichiana* formed compact groups, whilst the *T. mairei* samples formed a more scattered cluster. The 11 *T. mairei* samples from South China fell in the *T. mairei* cluster (Figure 3). Out of the 20 individuals collected from the Sindhupalchok district, Central Nepal, 18 fell inside the *T. wallichiana* group, but two individuals (RC1250, RC1251) collected on top of the ridge (in approximately 800 m distance from others) fell between the cluster of *T. contorta* and *T. wallichiana* in the scatter plot (Figure 3).

**Table 1:** Character ranks for the 27 morphological characters of 790 *Taxus* samples from the Hindu Kush-Himalaya and adjacent regions, determined by the product between the PCA descriptor axis values and proportion of variation for each axis.

Character	PC1	% var	Rank	PC2	% var	Rank	PC3	% var	Rank	Sum (%var)
Percent of variance		37.50			20.90			7.50		
*25*. Number of stomata bands*	0.268	0.101	5	0.171	0.036	12	0.056	0.004	11	0.140
*11*. Leaf arrangement*	0.291	0.109	1	0.133	0.028	17	0.002	0.000	25	0.137
*21*. Midrib shininess*	0.165	0.062	12	0.343	0.072	1	0.030	0.002	17	0.136
*23*. Margin shininess*	0.165	0.062	14	0.343	0.072	2	0.030	0.002	18	0.136
*9*. Leaf margin taper*	0.268	0.101	4	0.164	0.034	13	0.006	0.000	24	0.135
*10*. Leaf base symmetry*	0.287	0.108	2	0.132	0.028	18	0.000	0.000	27	0.135
*19*. Papillation on midrib*	0.163	0.061	15	0.336	0.070	3	0.049	0.004	12	0.135
*22*. Margin color*	0.165	0.062	13	0.334	0.070	4	0.026	0.002	19	0.134
*12*. Leaf apex symmetry*	0.267	0.100	6	0.141	0.029	15	0.011	0.001	22	0.130
*8*. Leaf curvature*	0.272	0.102	3	0.121	0.025	19	0.034	0.003	16	0.130
*7*. Length/Width ratio*	0.252	0.095	8	0.049	0.010	23	0.204	0.015	6	0.120
*27*. Bud scale persistence*	0.152	0.057	17	0.285	0.060	5	0.017	0.001	21	0.118
*5*. Leaf narrowest width*	0.222	0.083	10	0.034	0.007	24	0.289	0.022	5	0.112
*17*. Leaf midrib (abaxial)*	0.154	0.058	16	0.236	0.049	7	0.047	0.004	13	0.111
*6*. Leaf widest width*	0.231	0.087	9	0.006	0.001	27	0.290	0.022	4	0.110
*13*. Leaf apex shape*	0.263	0.099	7	0.029	0.006	25	0.042	0.003	14	0.108
*3*. Leaf shortest length	0.108	0.041	21	0.086	0.018	21	0.564	0.042	2	0.101
*16*. Leaf edges	0.104	0.039	23	0.263	0.055	6	0.060	0.005	10	0.098
*4*. Leaf longest length	0.098	0.037	24	0.073	0.015	22	0.595	0.045	1	0.097
*15*. Leaf texture	0.151	0.057	18	0.176	0.037	10	0.019	0.001	20	0.095
*26*. Stomata density	0.139	0.052	20	0.187	0.039	9	0.039	0.003	15	0.094
*2*. Leaf density	0.167	0.063	11	0.022	0.005	26	0.292	0.022	3	0.089
*20*. Midrib color	0.105	0.039	22	0.201	0.042	8	0.079	0.006	7	0.087
*1*. Leaf angle	0.150	0.056	19	0.117	0.024	20	0.061	0.005	9	0.085
*14*. Mucro	0.089	0.033	26	0.172	0.036	11	0.008	0.001	23	0.070
*18*. Leaf midrib (adaxial)	0.095	0.036	25	0.139	0.029	16	0.066	0.005	8	0.070
*24*. Margin width up to midrib	0.043	0.016	27	0.145	0.030	14	0.002	0.000	26	0.047

* indicates strong characters based on descriptor loading (in descending order).

The three distinct groups of samples observed in the 2D PCA scatter plot were found to be highly significant in the discriminant analysis (Wilks’ lambda = 0.003; F _6,787_ = 4628.199; *P*<0.001). The cross validated classification showed that overall 99.7% of the samples were correctly classified (i.e. all except RC1250 and RC1251). The test for equality of group means for each principle components was highly significant for PC1 (Wilks’ lambda = 0.004; F _2,784_ = 8548.611; *P*<0.000) and PC2 (Wilks’ lambda = 0.064; F _2,784_ = 5730.861; *P*<0.000), but not for PC3 (Wilks’ lambda = 0.998; F _2,784_ = 0.749.; *P* = 0.473) which suggested that the strong differences occurred along axes PC1 and PC2.

The contribution of individual characters in grouping the *Taxus* samples in the PCA scatter plot was evaluated from the descriptors loadings of the first three principle component axes (Table 1). For the yews from the HKH and adjacent regions, sixteen characters contributed strongly to the grouping of the samples. The remaining eleven characters (Table 1) were less powerful in this respect. Their removal greatly increased the proportion of variance in the first three coordinate axes (87%) of a PCA, and there was no difference in the pattern of specimen grouping in the PCA scatter plot (data not shown).

The character means of the three distinct PCA groups were found to be significantly different in the ANOVA (Table 2) and Chi-square test (Table 3). The differences were particularly high for the number of stomata bands (char. 25), leaf length/width ratio (char. 7), leaf apex shape (char. 13), leaf margin shininess (char. 23) and leaf arrangement (char. 11).

**Table 2:** Mean differences of continuous morphometric variables of 790 *Taxus* samples from the Hindu Kush-Himalaya and adjacent regions, clustering in three distinct groups suggested by the PCA. Means ± SD are given, as well as the *F* and *P* values of the ANOVA.

Characters	*T. contorta* (N = 333)	*T. mairei* (N = 60)	*T. wallichiana* (N = 397)	*F*	*P* value
2. Leaf density per 2 cm branch	11.95±1.291	10.00±1.683	10.52±1.251	129.108	0.000
3. Leaf Min. length (mm)	23.62±3.655	21.48±3.534	20.78±3.522	56.891	0.000
4. Leaf Max. length (mm)	30.67±4.584	27.72±4.648	27.33±4.670	47.599	0.000
5. Leaf Min. width (mm)	1.72±0.231	2.47±0.414	2.054±0.275	251.421	0.000
6. Leaf Max. width (mm)	2.00±0.244	2.94±0.500	2.54±0.427	269.689	0.000
7. Ratio (average)*	14.65±2.115	9.282±2.026	10.556±1.733	476.949	0.000
25. No of stomatal bands*	7.38±1.057	12.77±1.322	14.84±1.339	3279.340	0.000

* indicate strong characters. Tukey HSD *post hoc* test, only less significant comparisons are given: Leaf density - *T. mairei* to *T. wallichiana* (*P* = 0.011); Leaf min. length - *T. mairei* to *T. wallichiana* (*P* = 0.332); Leaf max. length - *T. mairei* to *T. wallichiana* (*P* = 0.815).

**Table 3:** Differences of discrete morphometric variables in the three distinct groups found among 790 *Taxus* samples from the Hindu Kush-Himalaya and adjacent region suggested by PCA.

Characters	*T. contorta* (N = 333)	*T. mairei* (N = 60)	*T. wallichiana* (N = 397)	Significance (χ^2^) (N = 790)°
*1*. Leaf angle	40°–80°	60°–80°	60°–90°	
*8*. Leaf curvature*	(0) 75%	(3) 69%	(3) 68%	(6, N) = 762.50, 0.000)
*9*. Leaf margin taper*	(0) 92%	(2) 65%	(3) 80%	(6, N) = 857.69, 0.000)
*10*. Leaf base symmetry*	(0) 97%	(1) 100%	(1) 100%	(2, N) = 750.12, 0.000)
*11*. Leaf arrangement*	(2) 100%	(1) 100%	(1) 100%	(2, N) = 787.00, 0.000)
*12*. Leaf apex symmetry*	(0) 98%	(1) 85%	(1) 95%	(2, N) = 657.40, 0.000)
*13*. Leaf apex shape *	(1) 78%	(3) 79%	(2) 84%	(8, N) = 1100.46, 0.000)
*14*. Mucro	(1) 81%	(0) 92%	(1) 79%	(2, N) = 149.89, 0.000)
*15*. Leaf texture	(1) 81%	(0) 82%	(1) 92%	(6, N) = 495.08, 0.000)
*16*. Leaf edges	(1) 99%	(2) 79%	(1) 96%	(4, N) = 595.18, 0.000)
*17*. Leaf midrib (abaxial)	(0) 96%	(2) 84%	(0) 87%	(4, N) = 419.51, 0.000)
*18*. Leaf midrib (adaxial)	(0) 100%	(0) 98%	(0) 69%	(4, N) = 132.44, 0.000)
*19*.Papillation on midrib*	(2) 100%	(0) 93%	(2) 100%	(4, N) = 724.90, 0.000)
*20*. Midrib color*	(0) 86%	(1) 100%	(0) 83%	(2, N) = 230.56, 0.000)
*21*. Midrib shininess*	(0) 100%	(1) 100%	(0) 100%	(2, N) = 787.00, 0.000)
*22*. Margin color*	(0) 100%	(1) 100%	(0) 99%	(2, N) = 734.72, 0.000)
*23*. Margin shininess*	(0) 100%	(1) 100%	(0) 100%	(2, N) = 787.00, 0.000)
*24*. Margin width up to midrib	(1) 85%	(2) 60%	(1) 87%	(4, N) = 108.35, 0.000)
*26*. Stomata density	(1) 82%	(1) 90%	(2)52%, (1)47%	(4, N) = 296.72, 0.000)
*27*. Bud scale persistence*	(1) 84%	(1) 88%	(2) 94%	(4, N) = 563.78, 0.000)

* indicate strong characters; character state in brackets with corresponding percentage of occurrence to the right;

°χ**^2^** (df, N = number of valid cases = 790) = Pearson Chi-Square value, *P* value).

### Molecular Analysis

The length of the ITS sequences of *T. contorta*, *T. wallichiana* and *T. mairei* was 1155, 1156 and 1167 base pairs (bp) respectively. The aligned ITS matrix was 1167 characters and contained 27 variable sites (Table S4). The two samples SL18 (RC1250) and SL19 (RC1251) from the Sindhupalchok district, Central Nepal, showed additive polymorphic bases where *T. contorta* and *T. wallichiana* consistently differed (Table S4). The length of the *trn*L-F sequences ranged from 833 to 881 bp among the three species. The aligned *trn*L-F matrix was 881 characters long and included 15 variable sites with two indel regions (Table S5). The two accessions SL18 (RC1250) and SL19 (RC1251) from the Sindhupalchok population had *trn*L-F sequences identical to *T. contorta.*

**Figure 4:**
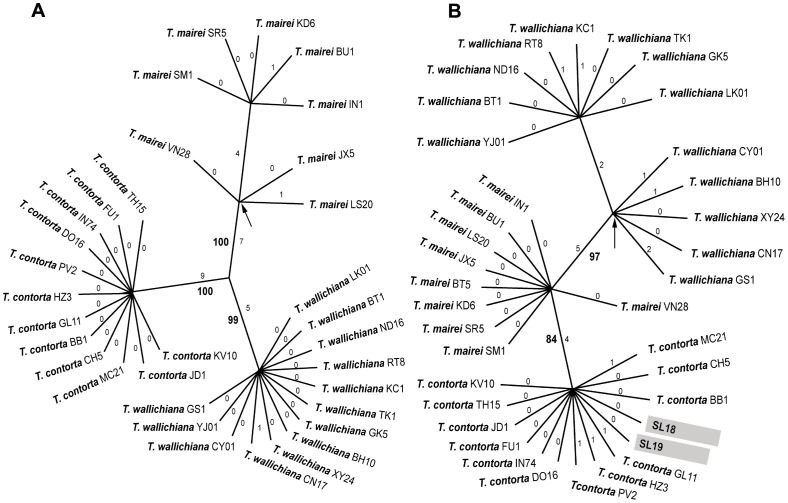
Maximum parsimony analysis. Unrooted cladogram based on **A.** 33 ITS sequences (one of three most parsimonious trees shown) and **B.** 36 *trn*L-F sequences (one of five most parsimonious trees shown) of the three *Taxus* species of the Hindu Kush-Himalaya and adjacent regions. Small numbers along the branches indicate branch lengths and numbers in bold indicate bootstrap values. Arrows indicate branches that collapse in the strict consensus trees. Accessions highlighted in light grey in the *trn*L-F tree indicate a different position from the ITS tree.

The MP analysis of the ITS sequences resulted in three most parsimonious trees of 28 steps length (CI = 0.929; RI = 0.991). All accessions corresponding to each of the three species formed strongly supported monophyletic clades with nine synapomorphies for samples of *T. contorta* (1.65% divergence) (100% bootstrap support, BS), five for *T. wallichiana* (1.21%) (99% BS) and seven for *T. mairei* (1.65%) (100% BS) (Figure 4A). While all *T. contorta* and all but one *T. wallichiana* ITS sequences were identical, those of *T. mairei* showed some diversity (Figure 4A, Table S4).

Five most parsimonious trees were found in the MP analysis of the *trn*L-F sequences, with a tree length of 21 steps (CI = 0.905; RI = 0.984). The *T. contorta* samples differed by four changes (0.49%) from *T. mairei* accessions (84% BS), while the *T. wallichiana* accessions were separated by five steps (0.75%) from those of *T. mairei* (97% BS) (Figure 4B). *Taxus mairei* and *T. contorta* accessions showed little intraspecific variation, but the *T. wallichiana* samples showed some variation falling into two clades (Figure 4B, Table S5).

### Ecological Niche Identity and Similarity

*Taxus contorta* is distributed in the Western Himalayas from eastern Afghanistan to Central Nepal and receives high winter rainfall from the Mediterranean West and relatively low summer rainfall. However, the eastern part of the *T. contorta* range stretches already into an area that receives low winter rainfall. This bimodal distribution of *T. contorta* with respect to rainfall in the driest and coldest quarters is clearly evident in the multivariate environmental space (Figure S1). *T. wallichiana* has a similar altitudinal range but occurs in the Eastern Himalayas from Central Nepal to Yunnan Province in China. Here it receives high summer rainfall due to the Indian monsoon, scarce winter rainfall, and experiences less annual temperature extremes (Figure S1, Table S6). Overall, *T. wallichiana* has a more restricted distribution in environmental space than *T. contorta* (Figure S1). *T. mairei* occurs at lower elevations and extends further South into the tropics, consequently experiencing warmer temperatures with less seasonality, higher precipitation but also more yearly variation in rainfall and less high winter rainfalls than *T. contorta* (Figure S2, Table S6).

The statistical tests developed by Broennimann et al. [36] show that the differences between the niches are significant (niche overlap *D* between *T. contorta* and *T. wallichiana* 0.223, *p*≤0.05; *T. contorta* and *T. mairei* 0.139, *p*≤0.05; and *T. wallichiana* and *T. mairei* 0.364, *p*≤0.05) (Figures S1–S3). Thus, the hypothesis of niche equivalency between any pair of the three species can be rejected. However, the niche similarity test which examines whether the observed overlap between niches in two ranges is different to simulated overlap when niches are picked at random from one of the ranges showed that in some cases the niches were more similar than expected by chance. For example, when the observed densities of occurrences in the *T. mairei* range are shifted at random, niche overlap was consistently lower than the observed niche overlap to *T. wallichiana* and *T. contorta* (Figures S1–S3). This is likely due to the fact that the *T. mairei* range encompasses a large amount of environmental variation from (coastal) tropical lowland areas to mountains within which the species is restricted to niches that (compared to this huge environmental background variation) are relatively similar to those of the other two *Taxus* species (mountain forests with comparatively low winter temperatures and high daily temperature extremes). When however, vice versa, the *T. contorta* niche is randomly resampled in its range and then compared to *T. mairei,* niche similarity is not significant. Two-way significant niche similarity only exists between *T. wallichiana* and *T. mairei.* These two species resemble each other for example in the fact that they both receive a relatively high amount of rainfall in the wettest quarter (monsoon) and in contrast to *T. contorta* no high rainfall in the driest/coldest quarters (Table S6). *T. wallichiana* and *T. contorta* also have some niche similarity but this is restricted to the eastern part of the *T. contorta* range. This significant niche similarity despite obvious differences in the niches occupied by the species outlined above is due to biases in the availability of environments for each of the species. Within the range of *T. wallichiana* for example there are no areas of high winter rainfall such as the ones occupied by *T. contorta*. As the statistical tests correct for the relative frequency of the environments, the comparison focuses on the environmental space available to both species (Central Nepal) and here the species indeed exhibit a similar distribution across the climate (Figure S1).

**Figure 5:**
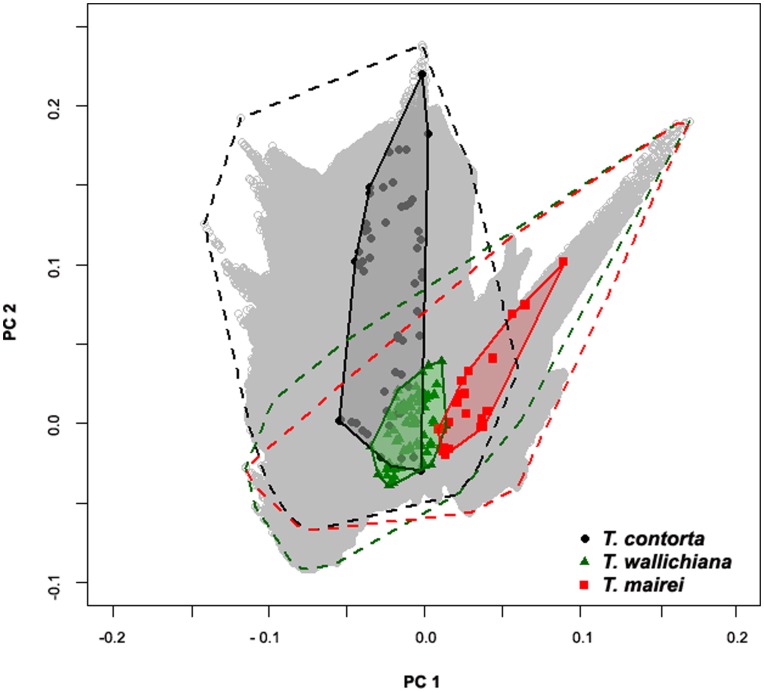
Principal component analysis (PCA) of bioclim variables and altitude. PCA plot of the environmental conditions in the study area based on 15 variables and altitude used in the analyses following Broennimann et al. (2012). The PCA is centered and scaled to unit variance. The polygons delineated with black, green and red dashed lines show the environmental space within the *T. contorta*, *T. wallichiana* and *T. mairei* ranges respectively. The grey, red and green shaded polygons are minimum convex polygons surrounding the environmental space of the occurrence records for the species, i.e. the occupied environmental space.

Figure 5 shows the climate space available to each of the species within a two-dimensional multivariate analysis (Principal Component Analysis) of environmental space. For maps of the first two principal components across the study area see figures S4 and S5. It is evident that although there are some overlaps the species largely occupy different climatic niches. However, the plot also shows that there are differences in the availability of environments for the species. Notably, large parts of the T. *contorta* range are situated in an environment that is not available to the other two species (Figures S4 and S5).

**Figure 6:**
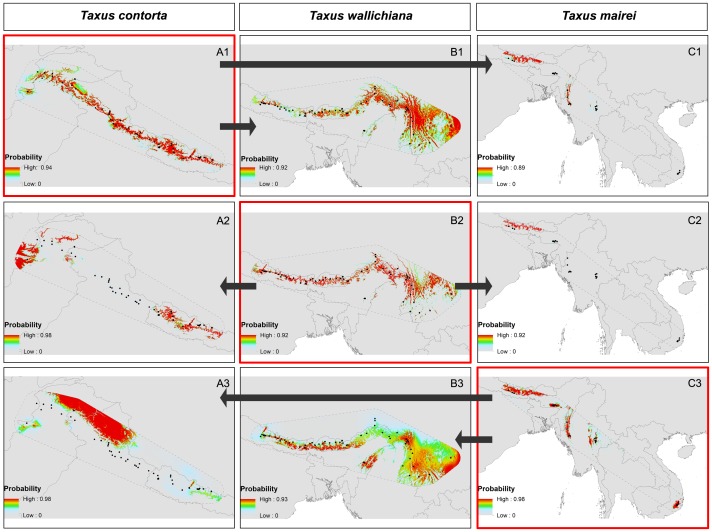
Ecological niche modeling. Comparison of species distribution models for **A**. *T. contorta*, **B**. *T. wallichiana* and **C**. *T. mairei*. The maps highlighted in red show the predictions for each species projected onto its own range: *T. contorta* (A1), *T. wallichiana* (B2), *T. mairei* (C3). The corresponding AUC values for these models are 0.948±0.023 SD (*T. contorta*), 0.958±0.018 SD (*T. wallichiana*) and 0.982±0.012 SD (*T. mairei*). The maps with a black margin show predictions for each species onto the range of another species: prediction of *T. wallichiana* (A2; AUC = 0.922) and prediction of *T. mairei* (A3; AUC = 0.774) onto the range of *T. contorta*; prediction of *T. contorta* (B1; AUC = 0.814) and prediction of *T. mairei* (B3; AUC = 0.786) onto the range of *T. wallichiana*; prediction of *T. contorta* (C1; AUC = 0.767) and prediction of *T. wallichiana* (C2; AUC = 0.932) onto the range of *T. mairei*. The AUC for these cross-model predictions are restricted to areas where the environment is within the range of the environments used for training the model.

Similarly, the cross-model comparisons showed that some parts of the species ranges can be predicted well using the model for another species whilst other parts are not well captured. For example, the eastern part of the range of *T. contorta* can be predicted by a model for *T. wallichiana* but this model falls short when it comes to predicting the western part of the range of *T. contorta* (Figure 6) (the AUC score however indicated a good predictability as it is restricted to the joint climate space in the eastern part of the range). *T. wallichiana* can be relatively well predicted from *T. contorta* although some over-prediction occurs as *T. contorta* generally occupies a larger niche space. *T. mairei* overall is poorly predicted from either of the two other yew species (only some parts of its northern range can be correctly predicted) as the latter two are restricted to higher altitudes where temperatures in the coldest quarter are not above 10°C whereas *T. mairei* can also occur at lower altitudes in areas with temperatures in the coldest quarter of up to 15°C (Table S6).

Additional predictions, whereby each model was forced to predict to a new area that was left out during training (Nepal), gave a similar picture: *T. mairei* was most poorly predicted and itself also produced poor models for the other two species, whereas *T. contorta* and *T. wallichiana* served as relatively good predictors for each other since Nepal is situated in the eastern part of the *T. contorta* range where its environmental niche is more similar to that of *T. wallichiana* (Figure S6). These models however partly reflect differences in the climate training data available to the models. *T. mairei* is better predicted by *T. contorta* and *T. wallichiana* as outside of Nepal *T. mairei* has few occurrences in a comparable environment. Consequently, when these are spared out in training model prediction for Nepal is poor.

### Taxonomic Aspects

Our analysis of morphological characters, nuclear and chloroplast sequences combined with climatic data and ecological niche modeling strongly suggests that three taxonomically distinct species of *Taxus* exist along the HKH and adjacent regions. An identification key exclusively for these three species based on the most informative characters indicated by our multivariate morphological analysis is provided below.

1a. Leaves mostly linear or straight; base symmetric; irregularly pectinate (arising from all direction along branchlets); stomatal bands 6–9 (10)…. **1.** ***T. contorta***
1b. Leaves lanceolate; sigmoid or falcate; base asymmetric; pectinate (arranged in two lateral rows on branchlets); stomatal bands 12–18…. **2**
2a. Bud scales less persistent or very few; leaves broadly lanceolate; midrib and leaf margin underneath shiny; apex acute-obtuse…. **2.** ***T. mairei***
2b. Bud scales persistent and dense; leaves narrowly lanceolate; midrib and leaf margin underneath not shiny, apex acuminate…. **3.** ***T. wallichiana***

1. ***Taxus contorta*** Griffith., Icon. Pl. Asiat. : t. 376. 1854.

**Type locality:** Afghanistan: Badakhashan, Kafiristan, Khasyah mts. (Lectotype: W. Griffith 5002, K).

**Synonyms:** *Taxus fuana* Nan Li & R.R. Mill (Holotype: Qingzang Expedition team 7032, PE).

**Diagnostic characters:** Evergreen tree to 14 m. Bud scales few ovoid, persistent at base of branchlets. Leaves arranged irregularly pectinate, leaves usually linear, equally wide throughout length, base cuneate, mostly symmetric, apex acute, midrib papillate, midrib and leaf margin underneath not shiny, loosely arranged,6–9(−10) stomatal bands, margin revolute - incurving when dried.

**Flowering/Fruiting:** Mid February-April/September-November.

**Habitat and Ecology:** 1900–3300(−3450)m, associated with *Abies*, *Acer*, *Pinus*, *Quercus*, *Rhododendron* and *Tsuga*, observed mostly along North, and Northwest facing slopes, common along wet gullies or inside forests. This species is relatively cold tolerant in comparison to other Himalayan yews.

**English Name:** To avoid confusion with other Himalayan yew species and to be consistent with Shah et al. [14] and Farjon [10], we regard this species as “*Western Himalayan Yew*”.

**Selected local names:** **Pakistan:** [*Barmi*, *Chodan*, *Srap*, *Thuna* [43], *Banrya* [44], *Burmi* [45]]; **NW India:** [*Thuner* [46], *Talispatra*, *Rakhal* [47]]; **Nepal:** [*Luit, Jemer Sing, Sanga Sing, Tongsa Sing, Dhengre, Lauth Salla, Kande Loti, Lota, Sigi, Thingre Salla, Talispatra* [7], [48]]; **China:** S Xizang [*Xizang Hong Dou Shan*, *Mi Ye Hong Dou Shan* [8]].

**Distribution:** **Afghanistan:** [Nuristan province and adjacent areas]; **Pakistan:** [Federally Administered Tribal Area (F.A.T.A.), North Punjab, Northwest Frontier Province (N.W.F.P.), Azad Kashmir)]; **NW India:** [Jammu & Kashmir, northern Himachal Pradesh, northern Uttaranchal Pradesh]; **Nepal:** [northern districts of western and Central Nepal (Northern slopes of Annapurna-Manaslu range)]; **China:** SW Xizang (Jilong valley).

**Conservation status:** Small sized populations are scattered irregularly along the western Himalayas. Highly threatened due to excessive commercial and local use. Overgrazing by livestock and slow regeneration and growth of the seedlings are additional threats [47], [49]. A few populations are well preserved in conservation areas.

**IUCN red list categories:** Global current threat status = EN A2cd; Proposed country wise current threat status (*Afghanistan* = CR A2cd, *China* = EN A2acd; B1ab (iii), *India (NW)* = EN A2cd, *Nepal* = EN A2acd, *Pakistan* = EN A2acd).

**Specimens examined:** see Table S1.

***Taxus mairei*** (Lemée & H. Léveillé) S.Y. Hu ex T.S. Liu, Illustr. Native & Introd. Lign. Pl. Taiwan 1∶16. 1960.

**Type locality:** China, NE Yunnan, Dongchuan Shi, (Holotype: E.E. Maire.n., E).

**Synonyms:** *Tsuga mairei* Lemée & H. Léveillé; *Taxus chinensis* (Pilger) Rehder var. *mairei* (Lemée & H. Léveillé) W.C. Cheng & L.K. Fu; *Taxus speciosa* Florin; *Taxus wallichiana* var. *mairei* (Lemée & H. Léveillé) L.K. Fu & Nan Li; *Taxus chinensis* var. *mairei* (Lemée & H. Léveillé) W.C. Cheng & L.K. Fu.

**Diagnostic characters:** Evergreen trees to 20 m. Bud scales at base of branchlets hardly persistent. Leaves pectinate, flat, broadly lanceolate, falcate or sigmoid, base asymmetric, apex acute-obtuse, midrib without papilla or rarely papillate below, mid rib and leaf margin underneath shiny.

**Flowering/Fruiting:** March-May/September-November.

**Habitat and Ecology:** 1500–2000 m along the Himalayas and adjacent regions but descending to 100 m in South China. In Central and East Nepal this species occurs in broadleaved forests along the Mahabharat range. It occurs in the Khasia Hills of Meghalaya, India.

**English Name:** “Maire’s Yew” or on the basis of its distribution this plant is named “South China Yew”.

**Local Names:** **Nepal:** [*Dhengre, Lauth Salla, Barme Salla, Pate Salla, Talispatra*]; **India:** [*Ksheh, Sehblei* [50], [51]]; **Bhutan:** [*Dhengrey Salla*]; **China:** [*Meili Hong Dou Shan*, *Bai Yin Shan*, *Shan Ba Shan*, *Nan Fang Hong Dou Shan* [8]], **Myanmar:** [*Kyauk-tinyu* [52]]; **Vietnam:** [*Thong Do Lam Dong, Thong Do La Dai, Thong Do Nam* [53]].

**Distribution:** **Nepal:** [Kavre and Sindhuli district]; **Bhutan:** [Central and Western Bhutan]; **India:** [Meghalaya (Khasia hills), at lower altitudes (<2000 m) in Nagaland and Manipur]; **Myanmar:** [low altitude (<2000 m) areas in the states of Shan and Chin]; **Vietnam:** [Lam Dong and Khanh Hoa province]; **China:** This species occurs abundantly in South China [13].

**Conservation status:** Along the HKH and adjacent regions, this species has a wide but scattered distribution with each population consisting of few individuals. Occurring at relatively low altitudes, many populations have been severely reduced due to diverse anthropological activities, such as logging, commercial collection, agriculture and expansion of settlement areas.

**IUCN red list categories:** Global current threat status = VU A2cd; Proposed country wise current threat status (*Bhutan* = CR A2cd, *China* = VU A2acd, *India (Meghalaya)* = CR A2cd, *Myanmar* = CR A2cd, *Nepal* = CR A2acd; B1ab (iii), *Vietnam* = CR A2cd; B1ab (iii)).

**Specimens examined:** see Table S1.

***Taxus wallichiana*** Zucc., Abh. Math.- Phys. Cl. Königl. Bayer. Akad. Wiss. 3: 803, t. 5. 1843.

**Type locality:** India (Eastern) (Lectotype: N. Wallich s.n., M).

**Synonyms:** *Taxus baccata* L. subsp. *wallichiana* (Zucc.) Pilger; *Taxus contorta* var. *mucronata* Spjut; *Taxus wallichiana* var. *yunnanensis* (W.C. Cheng & L.K. Fu) C.T. Kuan; *Taxus yunnanensis* W.C. Cheng & L.K. Fu; *Taxus wallichiana* var. *wallichiana* (Zucc.) W.C. Cheng & L.K. Fu; *Taxus virgata* Wall. ex Hook. f.

**Diagnostic characters:** Evergreen trees to 30 m. Bud scales at base of branchlets mostly persistent. Leaves pectinate, flat, lanceolate to narrowly lanceolate, gradually tapering toward apex, falcate or sigmoid, base asymmetric, apex acuminate, midrib papillate, midrib and leaf margin underneath not shiny, pale yellowish loosely-densely arranged (11)13–17(18) stomatal bands.

**Flowering/Fruiting:** January-April/September-November.

**Habitat and Ecology:** 1900–2700(−3300)m, in conifer forest or broad leaved mixed forests, associated with *Abies*, *Acer*, *Pinus*, *Quercus*, *Rhododendron*, *Tsuga*, bamboos and laurels. Observed mostly along Northeast and South facing slopes, common inside forests, along humid riversides and valleys. Grows in well-drained soil.

**English Name:** Considering its extensive distribution along the eastern Himalayas this species is referred to as “*Eastern Himalayan Yew*”.

**Selected local names:** **Nepal:** [*Silingi, Sanga Sing, Tongsa Sing, Dhengre, Dhayangre Salla, Lauth Salla, Salin, Barme Salla, La Swan, Sigi, Thingre Salla, Talispatra* [7], [48]]; **India:** [*Dhayngre Salla, Cheongbu, Kitangma, Talisa, Talispatra, Kazei Matang* [54], [55]]; **Bhutan:** [*Keyrang-shing, Kirang-shing, Dhengrey Salla* [56]]; **China:** [*Yunnan Hong Dou Shan*, *Xu Mi Hong Dou Shan* [8]]; **Myanmar:** [*Kyauk-tinyu* [52]].

**Distribution:** **Nepal:** [eastern part of Baglung district along the South of the Dhaulagiri and Annapurna range to East Nepal]; **Bhutan:** [entire northern part of the country]; **India:** [NE India including Nagaland and Manipur, mostly above 1900 m]; **Myanmar:** [northern parts of Sagaing division, Kachin and Shan states]; **China:** [South and Southeast Tibet (from the eastern border of Sikkim) to Southwest and West Yunnan].

**Conservation status:** Populations are sporadically distributed from Central Nepal eastwards along the eastern Himalayas. In most parts of its range it is threatened due to excessive commercial and local use, deforestation, overgrazing and slow regeneration.

**IUCN red list categories:** Global current threat status = EN A2cd; Proposed country wise current threat status (*Bhutan* = VU A2cd, *China* = EN A2acd, *India (E)* = EN A2cd, *Myanmar* = EN A2cd, *Nepal* = EN A2acd).

**Specimens examined:** see Table S1.

## Discussion

### Species Delimitation and Differentiation

The number of *Taxus* species and their boundaries in the HKH and adjacent regions has been uncertain with as few as two and as many as eight species recognized in more traditional, morphology based taxonomic treatments [8]–[10]. More recent studies [11], [13], [14], [27], have used a combination of molecular and morphological data from range wide collections to resolve some of this uncertainty and clearly delimit two species (*T. wallichiana* and *T. contorta*). In the present study three distinct species, *T. contorta*, *T. wallichiana* and *T. mairei*, were found to occur in the HKH and adjacent regions. Their strong molecular separation was underpinned by morphological data and by ecological differentiation. The most intriguing findings of our study were the clear separation of the distributions of the yews in the HKH and adjacent regions and the first report of *T. mairei* in this region, a species that has previously been considered to be endemic to the South of China [8], [13].

The taxonomy of *Taxus* in the HKH and adjacent regions has been considered difficult due to the very few reliable morphological characters available for diagnosing species [8], [10]. Of the 27 morphological characters used here, more than half (Table 1) appeared to be extremely useful to identify and differentiate the three species here. In contrast to previous taxonomic treatments on *Taxus* [8]–[10], our findings strongly oppose views of lack of morphological differences among yews in the HKH and adjacent regions. The three yew species were readily differentiated and identified using the combination of a few morphological characters: e.g. bud scales, leaf arrangement, shape of the leaf and leaf tips, and shininess of the midrib and leaf margin adaxially. These characteristics are recognizable in the field, even by non-professional botanists.

### Climatic Differentiation

Climatic factors are important for the distribution and adaptation of plants [16], thus they may be useful to discriminate morphologically similar species. Due to the East to West orientation of the mountain ranges of the Himalayas, even small latitudinal differences result in marked variations in rainfall patterns, snow lines and edaphic conditions between the western and eastern Himalayas [24], [57], [58]. The three yew species here not only had distinct geographic distributions but there were also differences in their ecological niches. We used a range of different tests to investigate niche identity and similarity between the yew species. Although some similarities exist these tests have shown that the niches are non-equivalent. This was confirmed by both ordination and species distribution modeling approaches, and despite differences in the relative frequencies of environments available to the species for which the tests corrected. Each of the species extends into other areas with different climates that are not occupied by any of the other species: for example, *T. contorta* into an area of high winter rainfall in the western Himalayas and *T. mairei* into an area of tropical high temperatures and rainfall in South East Asia. While *T. contorta* and *T. wallichiana* occur at high altitudes with low winter temperature, *T. mairei* can occur at lower altitudes (Figures 5, 6 and S1–S3). Due to the different climatic conditions in the western and eastern Himalayas and the effects of the monsoons the species experience different winter and summer precipitation; *T. contorta* grows on north-facing slopes in areas with mid latitude moderate westerly winter rainfall and low summer rainfall, while *T. wallichiana* evolved with the South Asian higher summer rainfall (Indian Ocean monsoon) in habitats with predominantly south-facing aspects. *Taxus mairei* habitats are characterized by a great seasonality with low winter and very high summer rainfall. These potentially differential adaptations to ecological factors may be important for the emergence or reinforcement of their distinct geographic distributions and lead to reproductive isolation. Climatic variables, such as rainfall and temperature have been proposed to be involved in driving ecological speciation in other plant species. Another noteworthy finding supported by the PCA scatter plots and ecological niche models was that *T. contorta* and *T. mairei* may have a broader ecological amplitude than *T. wallichiana*. It should be noted that our conclusions regarding the climate niches occupied by the species might fall short due to the insufficiently high resolution and density of the available climate data for an area as topographically heterogeneous as the Himalayas. Due to the low resolution of the climate data, differences in the niches occupied by the species are likely somewhat underestimated. Further work based on high resolution data that captures local microclimates and edaphic conditions are needed to understand the niches and niche evolution of the yews of HKH region.

### Geographic Distribution and Biogeographic Implications

*Taxus wallichiana* has historically been considered to be confined to the Himalayas, Southwest China and southern Vietnam. More recently, the World Checklist of Conifers [10], which is used as the International Union for the Conservation of Nature’s (IUCN) Global Red list of Threatened Species and the Convention on International Trade in Endangered Species (CITES), extended the distribution range of this species as far as Sulawesi and Sumatera in Indonesia. However, our results and a recent population genetic study of *T. wallichiana* [28], [33] drew clear boundaries, with *T. wallichiana* distributed from the West Yunnan Plateau and Northwest Yunnan along the eastern Himalayas (South and Southeast Tibet, Bhutan and Northeast India) into Central Nepal (the eastern part of Baglung District), and towards the South only along North-South stretching mountain ridges (>2000 m) between Myanmar and Northeast India. The distribution range of *T. contorta* is confined to the western Himalayas, extending from East Afghanistan along the West Himalayas (North Pakistan and Northwest India) eastwards into Central Nepal and South West Xizang (Jilong valley).

*Taxus contorta* and *T. wallichiana* were thought to overlap in Central Nepal [14]. However, our studies here found them distributed parallel but not overlapping along the North (*T. contorta*) and South (*T. wallichiana*) of the Annapurna-Manaslu range, and a hybrid zone, as suggested earlier [11], does not exist. Only a minimal contact point exists in the northern part of the Rasuwa and Sindhupalchok districts of Central Nepal. The two individuals (RC1250/SL18 - height: 5 m, CBH: 22 cm; RC1251/SL19 - height: 4.5 m, CBH: 20 cm) growing at the top of a ridge in the Sindhupalchok-Listi (eastern end of Central Nepal) within a *T. wallichiana* population had an intermediate morphology and their hybrid nature was confirmed by their additive ITS sequence positions (cf. [59]). Although we did not find populations of *T. contorta* nearby, the presence of *T. contorta* cpDNA in the plants suggest pollen-mediated gene flow from *T. contorta* (plastids are paternally inherited in *Taxus* [60]) and the likely presence of *T. contorta* plants nearby in the rugged mountains in the northern part of this district.

The predominantly allopatric distribution of yews in Central Nepal is similar to that of several other gymnosperm species [61]–[63]. The relatively recent geological uplift of the HKH over the last 30–10 million years [64] has opened new habitats and created opportunities for species range expansions. There is some evidence for an East to West migration of plants into Central Nepal [65], and *T. contorta* might have migrated from the western Himalayas and *T. wallichiana* from the eastern Himalayas into Central Nepal along a corridor of suitable habitats. This suggests the existence of a contact zone to form or transition zone to exist between phytogeographic regions as defined by Stearn in Central Nepal [66]. This boundary is pronounced in the region 83°E to 86° 30^’^E between the eastern and western Himalayas [66], [67]. Here we found support for the existence of such a zone, thought with *Taxus* species occurring in parallel, with little contact. This is evident from the detection of only two hybrid plants in one area, in the Sindhupalchok district of Central Nepal, representing the eastern most edge of the transition zone at 85° 52^’^E.

*Taxus mairei* was previously considered to be distributed in low to mid elevation montane forests in South China westward to Yunnan and West Sichuan, Southwest China [8], [13]. The western-most distribution of *T. mairei* was previously given for an isolated occurrence in Tengchong, West Yunnan, China, at elevations of 1780–1930 m [8]. Based on our results here, we found *T. mairei* is also discontinuously distributed within a relatively narrow altitudinal range of 1400–2000 m in southern Vietnam, Southeast and Central Myanmar, Northeast India, Bhutan and Nepal. Our results indicate that populations in southern Vietnam, formerly identified as *T. wallichiana* [68], should be referred to *T. mairei*. These seemingly disjunct occurrences may represent the remnants of a previous continuous distribution of this species in South and Southeast Asia. The furthest western distribution of *T. mairei* is now the Mahabharat range (1000–2500 m), the East-West stretching mountain belt immediately South to the lesser Himalayas in Nepal. In Nepal the distribution follows the mountain range South of Sunkoshi, Dudhkoshi and Tamor rivers. In the case of Bhutan and Meghalaya-India, we have to refer to data from herbarium specimens and their respective sequence data. However, detailed field work and population level sampling is necessary to understand the exact distribution of *T. mairei* here. As suggested by the MaxEnt ecological niche model, the lesser Himalayan part of Sikkim and Darjeeling are potential areas for *T. mairei*, but there are no records from these localities. *Taxus* growing in the northern part of Myanmar bordering India and China are *T. wallichiana* [27].

Correct species identification coupled with a comprehensive knowledge of each species’ distribution and ecological preferences are among the most basic requirements for effective conservation and for sustainable utilization [69]. Such data allows for more accurate assessments of the risk of extinction faced by each species at a local, regional and global scales and is crucial for the development of targeted conservation interventions and the implementation of international regulatory agreements such as CITES. The results from this study provide a reliable method of identification for yews within the HKH and adjacent regions, a clearer more detailed overview of the distribution of each species and preliminary data on each species’ ecological preferences. Our results also highlight the presence of small populations of *Taxus mairei* in several countries and indicate that *Taxus wallichiana* is much less widespread than previously thought [8]–[10].

Although Shah et al. [14] demonstrated the presence of *T. contorta* (as *T. fuana*) and *T. wallichiana* along the Himalayas, the extensive sampling in our study here provided the opportunity to delineate the distribution ranges for *T. contorta* and *T. wallichiana* in much greater detail, unraveling a distinct non-overlapping parallel distribution of the two species in Central Nepal, and the absence of a distinct hybrid zone contrary to a previous assumption [11]. The existence of a third species, *T. mairei*, was confirmed with a distinctly low altitude distribution in Nepal. The strongly spatially discrete occurrence of the three species is likely driven by landscape features of the HKH region and the differential climatic envelopes that were found to be very different for each species. Finally, the study demonstrated the advantages of the combination of robust sampling and molecular, morphological and climatic data to unambiguously differentiate and identify taxonomically difficult groups.

## Supporting Information

Figure S1 **Niche equivalency and similarity tests according to Broennimann et al. (2012) between** ***T. contorta*** **and** ***T. wallichiana*** **.** The top two graphs depict the species niches along the first two axes of a PCA calibrated on the entire environmental space in the two study areas. The bottom left graph shows to the contribution of each of the environmental variables to the ordination axes and the percentage of variance explained by these axes. The three histograms on the bottom right show the observed niche overlap (red line with diamond) and the simulated niche overlaps (grey bars) resampled for the niche identity test (upper histogram), and for the niche similarity test (two bottom histograms) cf. Warren et al. 2008 (Broennimann et al. 2012). The bottom left histogram (“Similarity 2->1”) compares observed niche overlap in the two ranges of *T. contorta* and *T. wallichiana* to simulated niche overlap when niches are drawn at random from the *T. wallichiana* range. In the bottom right histogram (“Similarity 1->2”) niches are drawn at random from the *T. contorta* range.
(TIFF)

Figure S2 **Niche equivalency and similarity tests according to Broennimann et al. (2012) between** ***T. contorta*** **and** ***T. mairei*** **.** The top two graphs depict the species niches along the first two axes of a PCA calibrated on the entire environmental space in the two study areas. The bottom left graph shows to the contribution of each of the environmental variables to the ordination axes and the percentage of variance explained by these axes. The three histograms on the bottom right show the observed niche overlap (red line with diamond) and the simulated niche overlaps (grey bars) resampled for the niche identity test (upper histogram), and for the niche similarity test (two bottom histograms) cf. Warren et al. 2008 (Broennimann et al. 2012). The bottom left histogram (“Similarity 2->1”) compares observed niche overlap in the two ranges of *T. contorta* and *T. mairei* to simulated niche overlap when niches are drawn at random from the *T. mairei* range. In the bottom right histogram (“Similarity 1->2”) niches are drawn at random from the *T. contorta* range.
(TIFF)

Figure S3 **Niche equivalency and similarity tests according to Broennimann et al. (2012)** ***between T. wallichiana*** **and** ***T. mairei*** **.** The top two graphs depict the species niches along the first two axes of a PCA calibrated on the entire environmental space in the two study areas. The bottom left graph shows to the contribution of each of the environmental variables to the ordination axes and the percentage of variance explained by these axes. The three histograms on the bottom right show the observed niche overlap (red line with diamond) and the simulated niche overlaps (grey bars) resampled for the niche identity test (upper histogram), and for the niche similarity test (two bottom histograms) cf. Warren et al. 2008 (Broennimann et al. 2012). The bottom left histogram (“Similarity 2->1”) compares observed niche overlap in the two ranges of *T. wallichiana* and *T. mairei* to simulated niche overlap when niches are drawn at random from the *T. mairei* range. In the bottom right histogram (“Similarity 1->2”) niches are drawn at random from the *T. wallichiana* range.
(TIFF)

Figure S4 **Principal component analysis of 15 bioclim variables and altitude.** Map showing the environmental variations among the three species of *Taxus* based on principal component 1 of a PCA.
(PDF)

Figure S5 **Principal component analysis of 15 bioclim variables and altitude**. Map showing the environmental variations among the three species of *Taxus* based on principal component 2 of a PCA.
(PDF)

Figure S6 **Ecological niche models of yews in Nepal**. Comparison of ecological niche models for **A**. *T. contorta*, **B**. *T. wallichiana* and **C**. *T. mairei* developed on training data where Nepal was left out, and predicted to Nepal. The corresponding AUC values for these models are 0.858 (*T. contorta*), 0.926 (*T. wallichiana*) and 0.672 (*T. mairei*). When *T. contorta* is predicted by *T. wallichiana* and *T. mairei* the corresponding AUC values are 0.835 and 0.8 respectively. When *T. wallichiana* is predicted by *T. contorta* and *T. mairei* the AUC values are 0.871 and 0.706. Finally, when *T. mairei* is predicted by *T. contorta* and *T. wallichiana* the AUC values are 0.723 and 0.776. It should be noted that these AUC scores partly reflect differences in the training data available to the models. *T. mairei* is better predicted by *T. contorta* and *T. wallichiana*, as outside of Nepal the species has few occurrences in a comparable environment.
(PDF)

Table S1 **Specimens examined**. List of specimens examined for the morphometric analysis and GenBank accession numbers of the samples used in the molecular analysis.
(PDF)

Table S2 **Morphological characters used**. Qualitative and quantitative characters used in the morphometric analyses (adopted from Möller et al. 2007). Character 11 shaded grey was modified in the present study.
(PDF)

Table S3 **Correlation matrix of 19 Bioclim variables and altitude**.
(PDF)

Table S4 **ITS sequence matrix**. Variable position of the nrDNA sequences (ITSLeu-4) of 33 accessions sampled across the distribution range of all three species of *Taxus*.
(PDF)

Table S5 ***trn*** **L-F sequence matrix.** Variable position of the cpDNA sequences (*trn*L-F) of 36 accessions sampled across the distribution range of all three species of *Taxus*.
(PDF)

Table S6 **Statistics of univariate ANOVA among** ***Taxus*** **of the Hindu Kush-Himalaya and adjacent regions for 19 bioclimatic variables.**
(PDF)
