# Supplementary figures and images for: Using Morphological, Molecular and Climatic Data to Delimitate Yews along the Hindu Kush-Himalaya and Adjacent Regions
Source: PLoS One. 2012 Oct 8;7(10):e46873. doi: 10.1371/journal.pone.0046873 (PMC3466193; doi:10.1371/journal.pone.0046873)

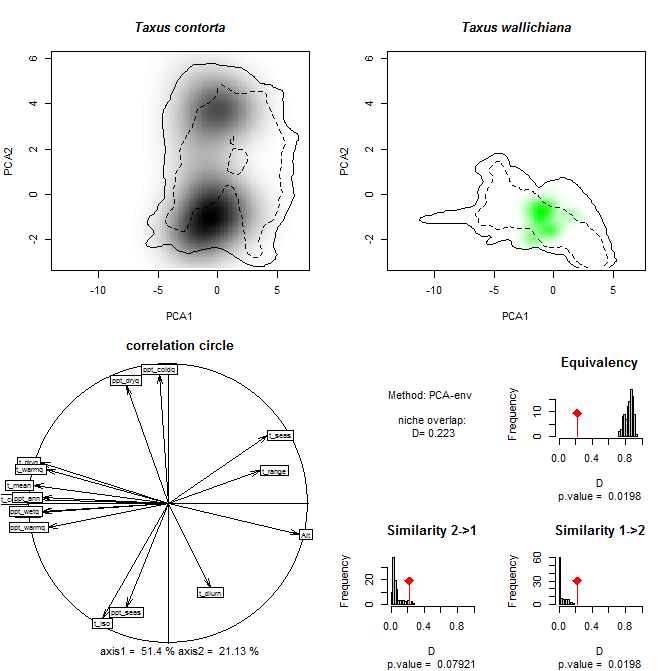

Supplement: Figure S1 — Niche equivalency and similarity tests according to Broennimann et al. (2012) between T. contorta and T. wallichiana . The top two graphs depict the species niches along the first two axes of a PCA calibrated on the entire environmental space in the two study areas. The bottom left graph shows to the contribution of each of the environmental variables to the ordination axes and the percentage of variance explained by these axes. The three histograms on the bottom right show the observed niche overlap (red line with diamond) and the simulated niche overlaps (grey bars) resampled for the niche identity test (upper histogram), and for the niche similarity test (two bottom histograms) cf. Warren et al. 2008 (Broennimann et al. 2012). The bottom left histogram (“Similarity 2->1”) compares observed niche overlap in the two ranges of T. contorta and T. wallichiana to simulated niche overlap when niches are drawn at random from the T. wallichiana range. In the bottom right histogram (“Similarity 1->2”) niches are drawn at random from the T. contorta range. (TIFF) [file pone.0046873.s001.tiff]

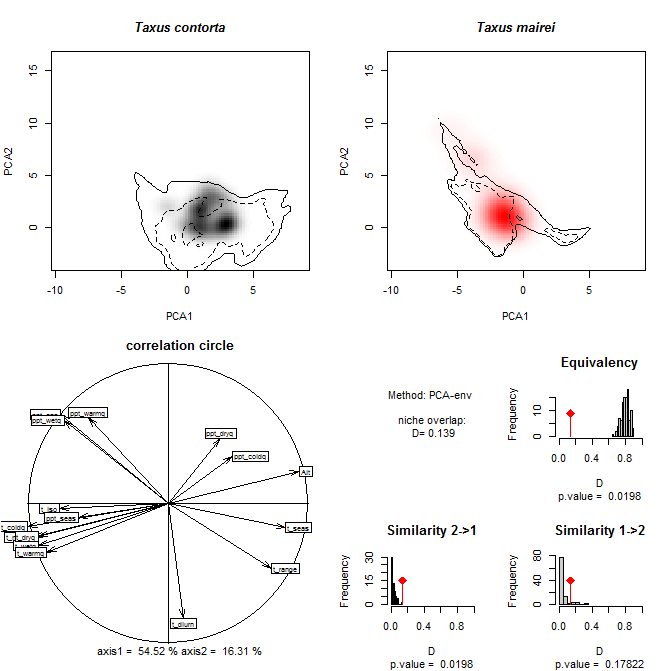

Supplement: Figure S2 — Niche equivalency and similarity tests according to Broennimann et al. (2012) between T. contorta and T. mairei . The top two graphs depict the species niches along the first two axes of a PCA calibrated on the entire environmental space in the two study areas. The bottom left graph shows to the contribution of each of the environmental variables to the ordination axes and the percentage of variance explained by these axes. The three histograms on the bottom right show the observed niche overlap (red line with diamond) and the simulated niche overlaps (grey bars) resampled for the niche identity test (upper histogram), and for the niche similarity test (two bottom histograms) cf. Warren et al. 2008 (Broennimann et al. 2012). The bottom left histogram (“Similarity 2->1”) compares observed niche overlap in the two ranges of T. contorta and T. mairei to simulated niche overlap when niches are drawn at random from the T. mairei range. In the bottom right histogram (“Similarity 1->2”) niches are drawn at random from the T. contorta range. (TIFF) [file pone.0046873.s002.tiff]

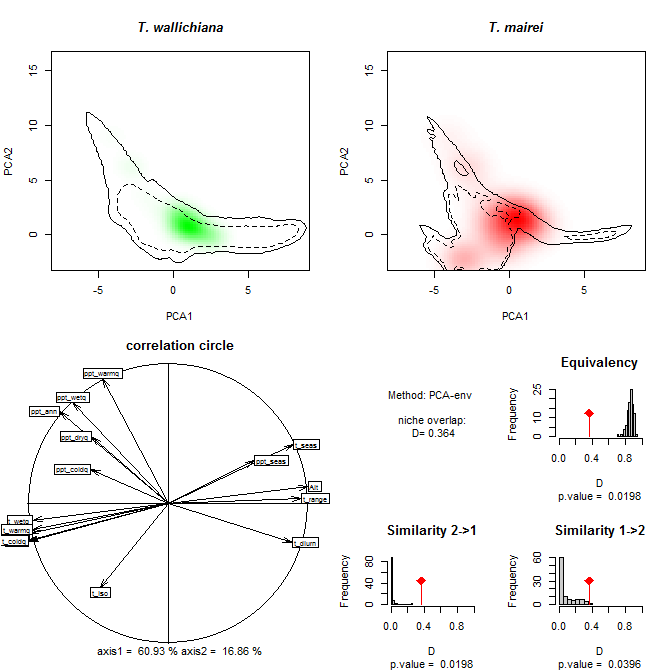

Supplement: Figure S3 — Niche equivalency and similarity tests according to Broennimann et al. (2012) between T. wallichiana and T. mairei . The top two graphs depict the species niches along the first two axes of a PCA calibrated on the entire environmental space in the two study areas. The bottom left graph shows to the contribution of each of the environmental variables to the ordination axes and the percentage of variance explained by these axes. The three histograms on the bottom right show the observed niche overlap (red line with diamond) and the simulated niche overlaps (grey bars) resampled for the niche identity test (upper histogram), and for the niche similarity test (two bottom histograms) cf. Warren et al. 2008 (Broennimann et al. 2012). The bottom left histogram (“Similarity 2->1”) compares observed niche overlap in the two ranges of T. wallichiana and T. mairei to simulated niche overlap when niches are drawn at random from the T. mairei range. In the bottom right histogram (“Similarity 1->2”) niches are drawn at random from the T. wallichiana range. (TIFF) [file pone.0046873.s003.tiff]

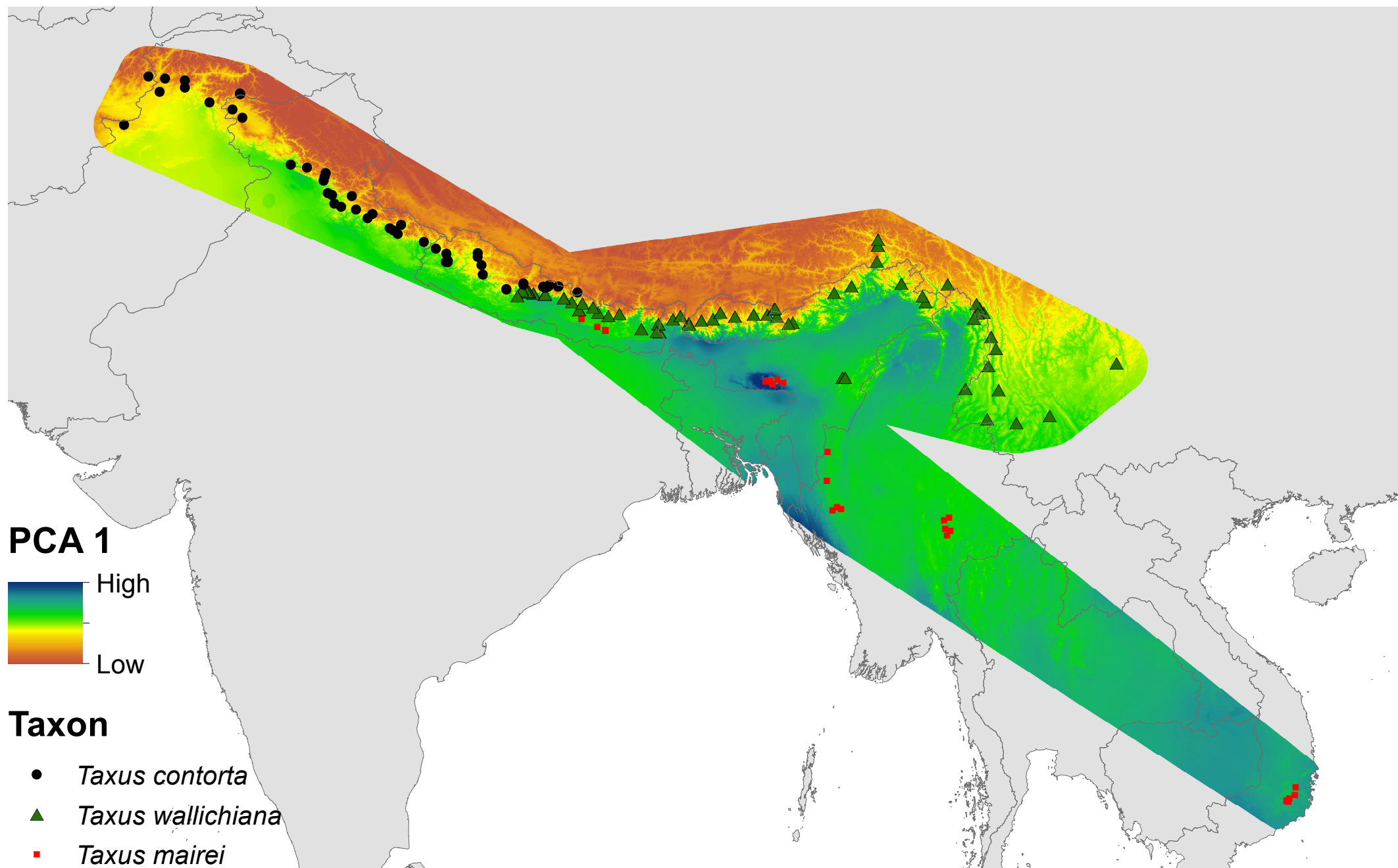

Supplement: Figure S4 — Principal component analysis of 15 bioclim variables and altitude. Map showing the environmental variations among the three species of Taxus based on principal component 1 of a PCA. (PDF) [file pone.0046873.s004.pdf]

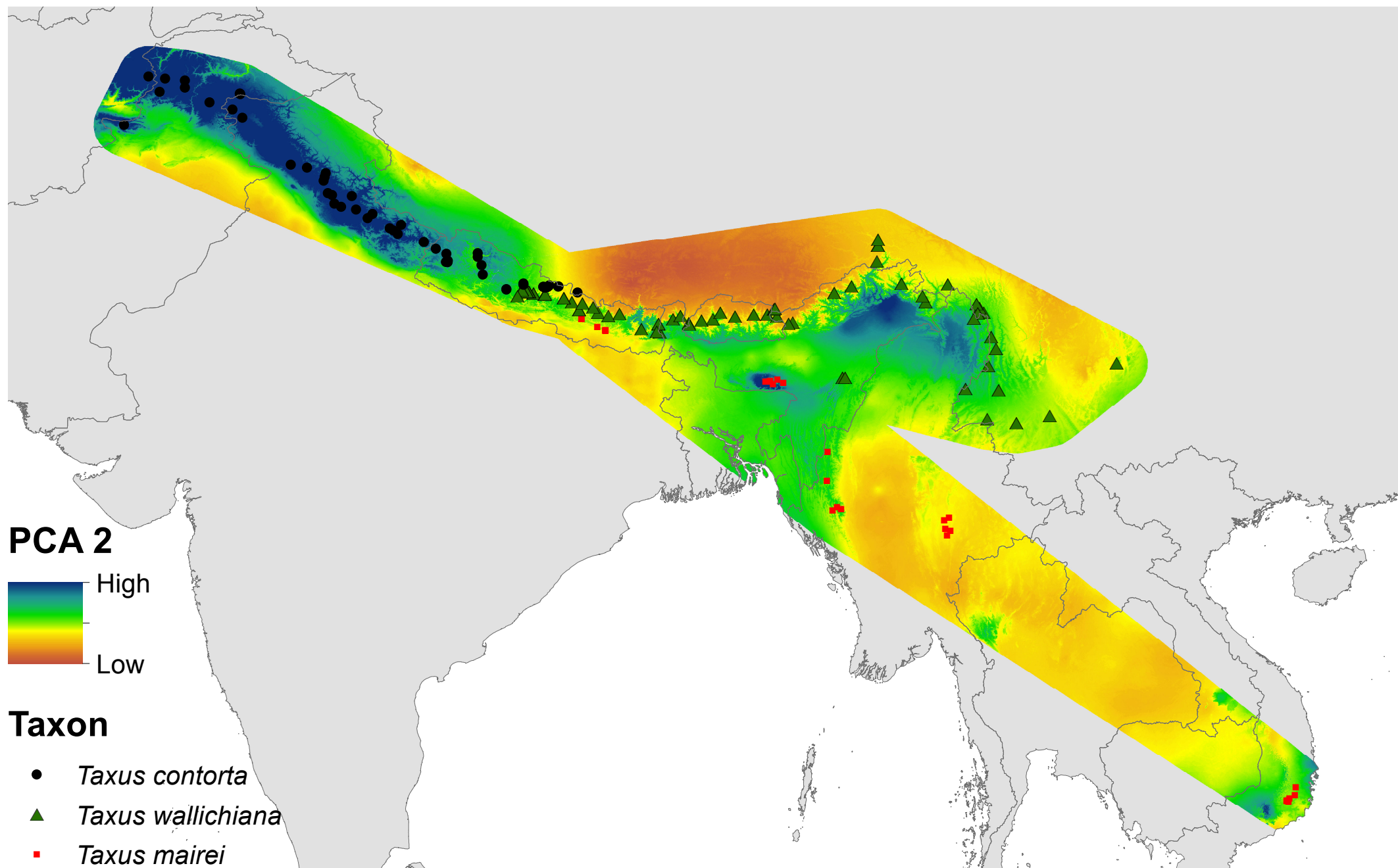

Supplement: Figure S5 — Principal component analysis of 15 bioclim variables and altitude. Map showing the environmental variations among the three species of Taxus based on principal component 2 of a PCA. (PDF) [file pone.0046873.s005.pdf]
