# Supplementary material for: Using Morphological, Molecular and Climatic Data to Delimitate Yews along the Hindu Kush-Himalaya and Adjacent Regions
Source: PLoS One. 2012 Oct 8;7(10):e46873. doi: 10.1371/journal.pone.0046873 (PMC3466193; doi:10.1371/journal.pone.0046873)
Supplement: Figure S6 — Ecological niche models of yews in Nepal. Comparison of ecological niche models for A. T. contorta, B. T. wallichiana and C. T. mairei developed on training data where Nepal was left out, and predicted to Nepal. The corresponding AUC values for these models are 0.858 (T. contorta), 0.926 (T. wallichiana) and 0.672 (T. mairei). When T. contorta is predicted by T. wallichiana and T. mairei the corresponding AUC values are 0.835 and 0.8 respectively. When T. wallichiana is predicted by T. contorta and T. mairei the AUC values are 0.871 and 0.706. Finally, when T. mairei is predicted by T. contorta and T. wallichiana the AUC values are 0.723 and 0.776. It should be noted that these AUC scores partly reflect differences in the training data available to the models. T. mairei is better predicted by T. contorta and T. wallichiana, as outside of Nepal the species has few occurrences in a comparable environment. (PDF) [file pone.0046873.s006.pdf]

Supplementary Figure S6

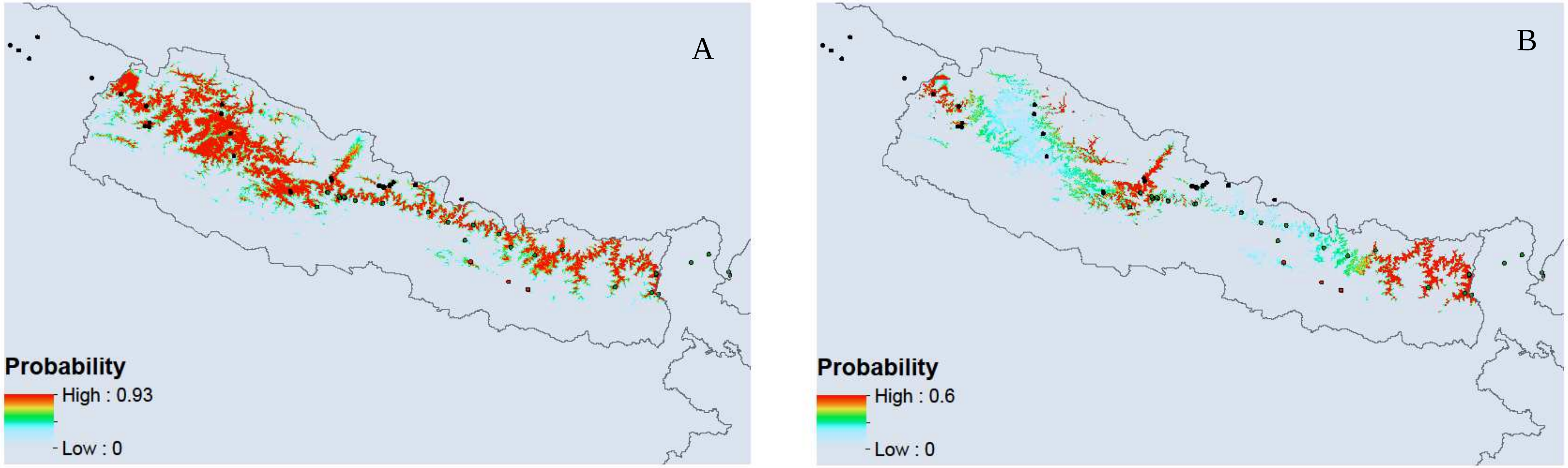

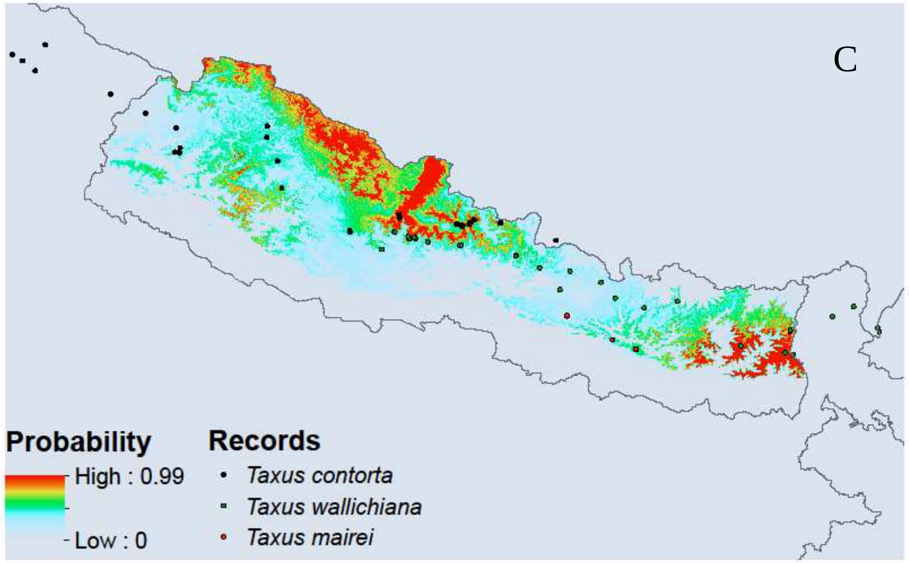

Figure S6. Ecological niche models of yews in Nepal. Comparison of ecological niche models for **A**. *T. contorta*, **B**. *T. wallichiana* and **C**. *T. mairei* developed on training data where Nepal was left out, and predicted to Nepal. The corresponding AUC values for these models are 0.858 (*T. contorta*), 0.926 (*T. wallichiana*) and 0.672 (*T. mairei*). When *T. contorta* is predicted by *T. wallichiana* and *T. mairei* the corresponding AUC values are 0.835 and 0.8 respectively. When *T. wallichiana* is predicted by *T. contorta* and *T. mairei* the AUC values are 0.871 and 0.706. Finally, when *T. mairei* is predicted by *T. contorta* and *T. wallichiana* the AUC values are 0.723 and 0.776. It should be noted that these AUC scores partly reflect differences in the training data available to the models. *T. mairei* is better predicted by *T. contorta* and *T. wallichiana*, as outside of Nepal the species has few occurrences in a comparable environment.
