# Supplementary material for: Using Morphological, Molecular and Climatic Data to Delimitate Yews along the Hindu Kush-Himalaya and Adjacent Regions
Source: PLoS One. 2012 Oct 8;7(10):e46873. doi: 10.1371/journal.pone.0046873 (PMC3466193; doi:10.1371/journal.pone.0046873)
Supplement: Table S1 — Specimens examined. List of specimens examined for the morphometric analysis and GenBank accession numbers of the samples used in the molecular analysis. (PDF) [file pone.0046873.s007.pdf]

Supplementary Table S1

Specimens collected at the population level are listed with population size but only a single citation is given here. Due to the conservation concerns of several fragmented, highly endangered populations, geographical coordinates have not been provided. For the herbarium specimens, information available on the label of each specimen is provided

Table S1. Specimens examined. List of specimens examined for the morphometric analysis and GenBank accession numbers of the samples used in the molecular analysis.

Country	Number of individuals	Locality	Altitude (m)	Voucher			Herbaria	GeneBank Accessions		Sequence Code
				Collection no.	Date	Collector		ITS	*trn*L-F	
T. contorta										
Afghanistan	1	Nuristan	2235	1979, L. Edelberg	6/7/1949	DerinElasoon	K	-	-	-
Pakistan	15	Azad Kashmir, Neelam Valley	2300	A25061	25/05/2005	A Shah	KUN	-	-	-
Pakistan	17	F.A.T.A. Kurram Valley	2439	A25324	15/08/2006	A Shah	KUN	JX680614	JX680646	KV10
Pakistan	15	F.A.T.A. Tirah	2000	A25309	30/06/2006	A Shah	KUN	JX680613	JX680645	TH15
Pakistan	15	N.W.F.P, Chitral	2500	A25275	4/07/2006	A Shah	KUN	JX680607	JX680636	CH5
Pakistan	15	N.W.F.P, Hazara	2200	A25208	06/05/2005	A Shah	KUN	JX680610	JX680641	HZ3
Pakistan	15	N.W.F.P, Palas Valley	2000	A25252	15/06/2006	A Shah	KUN	JX680611	JX680642	PV2
Pakistan	15	Punjab, JhikkaGali	2122	A25117	31/05/2005	A Shah	KUN	-	-	-
India	1	Bushreo Pass, Kulu, Punjab	2898	3119	8/11/1931	Walter Koelz	E	-	-	-
India	1	East Almora division, Kumaon	2745	2261	22/05/1933	Biskam	E	-	-	-
India	1	Himachal Pradesh, between Camp1 and Camp2 above Bathaad in the Great Himalayan National Park	2865	1912	09/1993	Howick, C & McNamara A	E	EF680236	EF680256	FU1
India	1	Kashmir, Kootihar valley	2430	85	04/12/1896	H. H. Johnston	E	-	-	-
India	1	Kashmir, Sind valley, 2-3 miles W of Sonamarg	2440	56/622	31/08/1956	O. Polunin	E	-	-	-
India	1	TrijugiNarainet, Garhwal	2242	8894	16/10/1938	Kirt Ram	E	-	-	-
India	20	Uttarakhand: Chamoli	2200	Yuhy-74	5/08/2007	Yu Hyaing	KUN	HM590974	HM591152	IN74
Nepal	23	Baglung, Bobang	2548	RC1587	12/05/2010	RC Poudel	KUN	JX680608	JX680637	BB1
Nepal	24	Darchula, Sitola	2664	RC1641	21/05/2010	RC Poudel	KUN	JX680612	JX680643	DO16
Nepal	18	Doti, Bichapani,	3090	RC1865	03/06/2011	RC Poudel	KUN	-	-	-
Nepal	12	Gorkha, Lihi	2907	RC1822	21/02/2011	RC Poudel	KUN	-	-	-
Nepal	20	Jumla, Depalgaun	2473	RC1652	25/05/2010	RC Poudel	KUN	JX680605	JX680644	JD1
Nepal	21	Manang, Ghattekhola	2690	RC1339	03/04/2010	RC Poudel	KUN	JX680606	JX680635	MC21
Nepal	20	Manang, Gho	2264	RC1370	04/04/2010	RC Poudel	KUN	-	-	-
Nepal	20	Manang, Temang	2643	RC1340	03/04/2010	RC Poudel	KUN	-	-	-
Nepal	20	Mustang	2447	RC1493	24/04/2010	RC Poudel	KUN	-	-	-
China	20	Jilong, Xizang	2395	GLM081630	14/09/2008	LM Gao & J Liu	KUN	JX680609	JX680640	GL11
China*	1	Xizhang, Jilong-Yin	2300	7032	20/7/1975	Qing-Zhang team	KUN	-	-	-
**Total specimens**	**325 +8=333**									

Country	Number of individuals	Locality	Altitude (m)	Voucher			Herbaria	GeneBank Accessions		Sequence Code
				Collection no.	Date	Collector		ITS	*trn*L-F	
T .mairei										
Nepal	14	Kavre	2030	RC1258	22/03/2010	RC Poudel	KUN	JX680615	JX680649	KD6
Nepal	20	Sindhuli	1756	RC1768	07/02/2011	RC Poudel	KUN	JX680617	JX680647	SM1
Nepal	5	Sindhuli	1450	RC1803	09/02/2011	RC Poudel	KUN	JX680616	JX680648	SR5
Bhutan	1	Between Punakha and Tashitang	1500	7368	10/3/1991	J.R.I. Wood	E	-	JX680650	BT5
India	1	Khasia, Kala Panu?,	1524	1337	27/06/1950	Hooker	K	-	-	-
India	1	Maophlang, Khasia	1800	38308	16/06/1885	Clarke	E	JX680618	JX680654	IN1
India	1	Nunghuai, Khasia hills	1524	1885	1/11/1885	C. Mann	K	-	-	-
Myanmar	1	Mindat District, Hilawng ridge	1116	1983	7/11/1962	U. Mg Gale	E	JX680619	JX680653	BU1
Myanmar	1	West Central: Esakan	1952	21901	31/03/1956	F. Kingdon Ward	K	-	-	-
Vietnam	1	Lam Dong, Don Duong Distr., Ho Tien, Lake of Angels, first site	1430	162	8/9/2001	M. F. Gardner, P. Thomas, N. D. T. Luu, & N. V. Chiet	E	-	-	-
Vietnam	1	Lam Dong, DucTrong Distr., Nui Voi, XaHiep An (Elephant Mountain)	1500	200	17/09/2001	P. Thomas, N. D. T. Luu, & N. V. Chi	E	-	-	-
Vietnam	1	Lam Dong, DucTrong Distr., XaHiep An (Elephant Mountain)	1420	64	31/08/2001	M. F. Gardner, P. Thomas, N. D. T. Luu, & N. V. Chiet	E	-	-	-
Vietnam	1	Lam Dong, Thanh Pho Dalat, XaXuanTho (commune), XuanTho station	1227	39	29/08/2001	M. F. Gardner, P. Thomas, N. D. T. Luu, & N. V. Chiet	E	JX680620	JX680655	VN28
China	1	Fujian, Fuqing	707	ZHXM0506141	07/08/2005	Z Xue-Mei	KUN	-	-	-
China	1	Guangxi, Jin Xiu	1002	GLM 05-960	9/12/2005	LM Gao	KUN	-	-	-
China	1	Guizhou, Nayong	1580	MMO-03-417	19/9/2003	M Moller	KUN	-	-	-
China	1	Guizhou, Furongba	950	MMO-03-403	17/9/2003	M Moller	KUN	JX680622	JX680652	LS20
China	1	Guizhou, Guochang	790	MMO-03-370	17/9/2003	M Moller	KUN	-	-	-
China*	1	Yunnan (?) (Nord)	750	s.n	x-5-19xxx	E.E.Maire	E	-	-	-
China	1	Yunnan (Nord), Tie-Chang-Keou	650	131 (?)	x-9-19xxx	E.E.Maire	KUN	-	-	-
China	1	Yunnan (Nord), Tie-Tchang-Keow	700	s.n. (acc. No.129)	x-9-19xxx	E.E.Maire	KUN	-	-	-
China	1	Yunnan, Ganhe village	1300	GLM-07647	20/05/2007	LM Gao	KUN	-	-	-
China	1	Yunnan, Weixin	1326	GLM-07614	20/05/2007	LM Gao	KUN	-	-	-
China	1	Zhejiang, Linan	349	ZHXM050696	30/06/2005	Z Xue-Mei	KUN	JX680621	JX680651	JX5
**Total specimens**	**39+21=60**									
T. wallichiana										
Nepal	20	Baglung	2328	RC1571	10/05/2010	RC Poudel	KUN	JX680629	JX680663	BH10
Nepal	14	Dolakha, Suspachhemawati	2340	RC1215	15/03/2010	RC Poudel	KUN	-	-	-
Nepal	20	Gorkha	2170	RC1287	27/03/2010	RC Poudel	KUN	JX680628	JX680661	GK5
Nepal	20	Kaski, Lalka danda	2280	RC1401	19/04/2010	RC Poudel	KUN	JX680626	JX680659	KC1
Nepal	24	Kaski, Ghandruk	2210	RC1431	22/04/2010	RC Poudel	KUN	-	-	-
Nepal*	1-	Kathmandu, Shivapuri	2400	6054 (a), (sheet 1)	25/12/1904	Wall. Cat.	K	-	-	-
Nepal	20	Kathmandu, Shivapuri	2450	RC1071	27/02/2010	RC Poudel & LM Gao	KUN	-	-	-
Nepal	7	Kavre	2050	RC1266	22/03/2010	RC Poudel	KUN	-	-	-
Nepal	20	Lamjung, Bhujung	1950	RC1834	27/02/2011	RC Poudel	KUN	-	-	-
Nepal	20	Myagdi	2537	RC1523	30/04/2010	RC Poudel	KUN	-	-	-
Nepal	24	Myagdi, Ghodepani	3050	RC1467	23/04/2010	RC Poudel	KUN	-	-	-

Country	Number of individuals	Locality	Altitude (m)	Voucher			Herbaria	GeneBank Accessions		Sequence Code
				Collection no.	Date	Collector		ITS	*trn*L-F	
Nepal	4	Ramechhap, Deurali	2906	RC1229	18/03/2010	RC Poudel	KUN	-	-	-
Nepal	19	Rasuwa	2383	RC1203	12/03/2010	RC Poudel	KUN	JX680625	JX680658	RT8
Nepal	20	Sankhuwasabha, Tamaphok	2799	RC1740	02/02/2011	RC Poudel	KUN	-	-	-
Nepal	4	Sindhupalchok, Helambu	2502	RC1818	12/02/2011	RC Poudel	KUN	-	-	-
Nepal	18	Sindhupalchok, Listi	2370	RC1233	21/03/2010	RC Poudel	KUN	-	-	-
Nepal	20	Solukhumbu	2623	ND2	9/11/2005	RC Poudel	E	JX680624	JX680657	ND16
Nepal	14	Taplejung	2945	RC1683	04/06/2010	RC Poudel	KUN	JX680627	JX680660	TK1
India	1	Barail range, Naga hills	2900	7755	23/12/1927	Kingdon ward	K	-	-	-
India	1	Bengal, Batassi	1981	5D	4/6/1960	J.M. Cowan	E	-	-	-
India	1	Chiban, Assam	2000	8090	11/4/1928	Kingdon ward,	K	-	-	-
India	1	Delei valley, Assam	2743	8594	26/08/1928	Kingdon ward,	K	-	-	-
India	1	Jakpho, Naja Hills	2590	41238B	25/10/1885	C.B. Clarke	K	-	-	-
India	1	Manipur, Sirohifarar	2130	6493	-	G Watt	E	-	-	-
India	1	Manipur, on the eastern frontier of India, Jopvo	2440	6208	09/02/1882	G Watt	K	-	-	-
India	1	Manipur, on the eastern frontier of India, Seriphar(s)i	3050	5955	17/01/1882	G Watt	K	-	-	-
India	1	Manipur, Sriobinfurar, on the eastern frontier of India	2135	6493	13/04/1882	G Watt	K	-	-	-
India	1	Sikkim, Tonglo	2743	7458	11/1879	J.S. Gamble	K	-	-	-
India	1	Sikkim, Tonglo	2895	2674	11/1874	J.S. Gamble	E	-	-	-
India	1	West Bengal, Darjeeling	2270	1119	5/8/1992	D.G.Long et al.	E	-	-	-
India	1	West Bengal, Singalila Range	2562	148	15/05/1965	F.de Vos&E.G.Corbett	E	-	-	-
Bhutan	1	Dochu-La-Pass	3300	7349	24/03/ 1983	H. Ern	E	-	-	-
Bhutan	1	Thimpu district, summit of Dochong La	3110	4417	13/04/1982	Grierson & Long	E	JX680623	JX680656	BT1
Bhutan	1	Thimpu district, valley above Taba	2520	999	13/05/1979	Grierson & Long	E	-	-	-
Bhutan	1	Tunle La near KingaRapden	3352	18672	10/4/1949	F. Ludlow, G. Sherriff & G. Taylor	E	-	-	-
China	11	Xizang, Bomi	2570	GLM-082079	27/09/2008	LM Gao	KUN	-	-	-
China	12	Xizang, Chayu	2650	GLM-082127	29/09/2008	LM Gao	KUN	JX680632	JX680662	CY01
China	11	Xizang, Cuona	2820	GLM-081809	23/09/2008	LM Gao	KUN	JX680631	JX680665	CN17
China	12	Xizang, Yadong	2700	GLM-081728	19/09/2008	LM Gao	KUN	JX680630	JX680664	XY24
China	21	Yunnan, Gongshan	1960	GLM-2301	17/8/2003	LM Gao	KUN	EF680253	EF680273	GS1
China	10	Yunnan, Lushui	2900	Liuj-09291	20/03/2009	J Liu	KUN	JX680634	JX680666	LK01
China	12	Yunnan, Yingjiang	2612	Liuj-08041	14/12/2009	J Liu	KUN	JX680633	JX680667	YJ01
Nepal	1	Sindupalchok, Listi **(hybridº)**	2558	RC1250	21/03/2010	RC Poudel	KUN	-	JX680638	SL18
Nepal	1	Sindupalchok, Listi **(hybridº)**	2558	RC1251	21/03/2010	RC Poudel	KUN	-	JX680639	SL19
**Total specimens**	**379+18=397**									

Total samples 790

* Type specimen, º *T. wallichiana* × *T. contorta*
