# Supplementary material for: Using Morphological, Molecular and Climatic Data to Delimitate Yews along the Hindu Kush-Himalaya and Adjacent Regions
Source: PLoS One. 2012 Oct 8;7(10):e46873. doi: 10.1371/journal.pone.0046873 (PMC3466193; doi:10.1371/journal.pone.0046873)
Supplement: Table S2 — Morphological characters used. Qualitative and quantitative characters used in the morphometric analyses (adopted from Möller et al. 2007). Character 11 shaded grey was modified in the present study. (PDF) [file pone.0046873.s008.pdf]

Supplementary Table S2

Table S2. Morphological characters used. Qualitative and quantitative characters used in the morphometric analyses (adopted from Möller et al. 2007). Character 11 shaded grey was modified in the present study.

Character number	State	Type	Coding	State
Leaf characters				
1	Angle	Discrete	0	90-100^0
			1	90^0
			2	80-90^0
			3	70-90^0
			4	70-80^0
			5	60-80^0
			6	60-70^0
			7	50-70^0
			8	40-50^0
			9	40-70^0
2	Density	Continuous	Number of leaves on 2-cm branch section	
3	Shortest length	Continuous	mm	
4	Longest length	Continuous	mm	
5	Narrowest width	Continuous	mm	
6	Widest width	Continuous	mm	
7	Length/width ratio	Continuous	Average lengths/widths	
8	Curvature	Discrete	0	Straight
			1	Some falcate, some straight
			2	Falcate
			3	Sigmoid
9	Margin taper	Discrete	0	Parallel
			1	Some parallel, some not
			2	Parallel to 2/3-3/4 from base
			3	Lanceolate (from base ornear 1/2 tapered)
10	Base symmetry	Discrete	0	Yes
			1	No
11	Leaf arrangement	Discrete	0	Spiral
			1	Pectinate
			2	Irregularly pectinate
12	Apex symmetry	Discrete	0	Yes
			1	No
13	Apex shape	Discrete	0	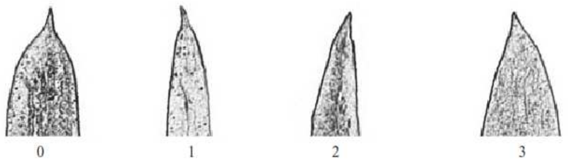
			1	
			2	
			3	
14	Mucro	Discrete	0	Indistinct
			1	Distinct
15	Texture	Discrete	0	Thin
			1	Medium
			2	Thick
16	Edges	Discrete	0	Flat
			1	Revolute
			2	Incurving to revolute
17	Mid rib appearance (abaxial)	Discrete	0	Elevated
			1	Level
			2	Sunken
18	Mid rib appearance (adaxial)	Discrete	0	Elevated
			1	Level
			2	Sunken
19	Papillation on mid rib	Discrete	0	Absent

Character number	State	Type	Coding	State
			1	Scattered
			2	Dense
20	Mid rib color	Discrete	0	Same
			1	Different
21	Mid rib shininess	Discrete	0	No
			1	Yes
22	Margin color	Discrete	0	Same
			1	Different
23	Margin shininess	Discrete	0	No
			1	Yes
24	Margin width up to mid rib	Discrete	0	Narrower
			1	Equal
			2	Broader
25	Number of stomatal bands	Continuous	Mean of five counts	
26	Stomata density in longitudinal direction	Discrete	0	Not dense (more thandiameter of stoma)
			1	Dense (less than diameter ofstoma)
			2	Very dense (almosttouching)
Bud Character				
27	Bud scale persistence	Discrete	0	None
			1	Some
			2	Most
