# Supplementary material for: Using Morphological, Molecular and Climatic Data to Delimitate Yews along the Hindu Kush-Himalaya and Adjacent Regions
Source: PLoS One. 2012 Oct 8;7(10):e46873. doi: 10.1371/journal.pone.0046873 (PMC3466193; doi:10.1371/journal.pone.0046873)
Supplement: Table S3 — Correlation matrix of 19 Bioclim variables and altitude. (PDF) [file pone.0046873.s009.pdf]

Supplementary Table S3

Table S3. Correlation matrix of 19 Bioclim variables and altitude

**Bioclim variables**	**Code**	**Altitide**	**BIO1**	**BIO2**	**BIO3**	**BIO4**	**BIO5**	**BIO6**	**BIO7**	**BIO8**	**BIO9**	**BIO10**	**BIO11**	**BIO12**	**BIO13**	**BIO14**	**BIO15**	**BIO16**	**BIO17**	**BIO18**	**BIO19**
Altitude	Alt		-0.97	0.42	-0.36	0.59	-0.95	-0.93	0.54	-0.96	-0.91	-0.98	-0.93	-0.54	-0.52	-0.20	-0.05	-0.53	-0.26	-0.27	-0.13
Annual Mean Temperature - Bio1	t_mean			-0.38	0.51	-0.72	0.95	0.98	-0.63	0.95	0.97	0.98	0.99	0.56	0.56	0.15	0.11	0.56	0.24	0.25	0.13
#**Mean Diurnal Range (Mean of monthly (max temp - min temp)) - Bio2**	t_diurn				-0.10	0.49	-0.19	-0.51	0.72	-0.28	-0.38	-0.30	-0.42	-0.64	-0.49	-0.60	0.55	-0.52	-0.63	-0.49	-0.28
#**Isothermality (BIO2/BIO7) (x 100) - Bio3**	t_iso					-0.85	0.31	0.60	-0.73	0.40	0.54	0.35	0.62	0.47	0.44	-0.08	0.09	0.44	0.04	0.21	0.18
Temperature Seasonality (standard deviation x100) - Bio4	t_seas						-0.49	-0.82	0.94	-0.57	-0.74	-0.55	-0.82	-0.70	-0.65	-0.16	0.04	-0.66	-0.27	-0.39	-0.20
Max Temperature of Warmest Month- Bio5								0.87	-0.37	0.93	0.91	0.99	0.89	0.36	0.40	0.05	0.22	0.39	0.11	0.05	0.07
Min Temperature of Coldest Month - Bio6									-0.77	0.90	0.96	0.92	0.99	0.64	0.62	0.22	0.02	0.62	0.32	0.31	0.19
Temperature Annual Range (BIO5-BIO6) - Bio7	t_range									-0.50	-0.65	-0.47	-0.74	-0.76	-0.66	-0.36	0.25	-0.67	-0.46	-0.53	-0.27
Mean Temperature of Wettest Quarter - Bio8	t_wetq										0.87	0.96	0.91	0.47	0.47	0.12	0.17	0.48	0.18	0.26	0.06
Mean Temperature of Driest Quarter - Bio9	t_dryq											0.93	0.97	0.55	0.56	0.14	0.10	0.56	0.24	0.20	0.18
Mean Temperature of Warmest Quarter - Bio10	t_warmq												0.93	0.44	0.46	0.13	0.15	0.46	0.19	0.16	0.10
#**Mean Temperature of Coldest Quarter - Bio11**	t_coldq													0.62	0.61	0.16	0.09	0.61	0.25	0.29	0.16
Annual Precipitation - Bio12	ppt_ann														0.93	0.37	-0.14	0.97	0.48	0.74	0.32
Precipitation of Wettest Month - Bio13																0.19	0.11	0.99	0.30	0.61	0.36
Precipitation of Driest Month - Bio14																	-0.54	0.23	0.93	0.42	0.25
Precipitation Seasonality (Coefficient of Variation) - Bio15	ppt_seas																	0.06	-0.53	-0.25	-0.13
#**Precipitation of Wettest Quarter - Bio16**	ppt_wetq																		0.34	0.67	0.32
#**Precipitation of Driest Quarter - Bio17**	ppt_dryq																			0.49	0.29
#**Precipitation of Warmest Quarter - Bio18**	ppt_warmq																				0.05
#**Precipitation of Coldest Quarter - Bio19**	ppt_coldq																				

#Uncorrelated variables used to develop ecological niche model for each yew species distributed in HKH and adjacent regions.

Altitude, Bio1, Bio2, Bio3, Bio4, Bio7, Bio8, Bio9, Bio10, Bio11, Bio12, Bio15, Bio16, Bio17, Bio18 and Bio19 were used to compare ecological niches among the three species following Broennimann et al. (2012).
