# Supplementary material for: Using Morphological, Molecular and Climatic Data to Delimitate Yews along the Hindu Kush-Himalaya and Adjacent Regions
Source: PLoS One. 2012 Oct 8;7(10):e46873. doi: 10.1371/journal.pone.0046873 (PMC3466193; doi:10.1371/journal.pone.0046873)
Supplement: Table S4 — ITS sequence matrix. Variable position of the nrDNA sequences (ITSLeu-4) of 33 accessions sampled across the distribution range of all three species of Taxus. (PDF) [file pone.0046873.s010.pdf]

Supplementary Table S4

Table S4. ITS sequence matrix. Variable position of the nrDNA sequences (ITSLeu-4) of 33 accessions sampled across the distribution range of all three species of *Taxus*.

Species	Country/Locality/Code	Positions																										
		**118**	**135**	**139**	**147**	**151**	**197**	**216**	**289**	**294**	**341**	**361**	**372**	**387**	**399**	**455**	**474**	**536**	**544**	**550**	**559**	**661**	**731**	**736**	**1026**	**1084**	**1089**	**1144**
T. contorta	Pakistan: Kurram Valley, KV10	T	T	T	T	T	C	T	-	T	T	C	T	C	C	C	T	A	A	G	T	T	G	A	T	T	G	T
	Pakistan: Tirah, TH15	.	.	.	.	.	.	.	-	.	.	.	.	.	.	.	.	.	.	.	.	.	.	.	.	.	.	.
	Pakistan: Chitral, CH5	.	.	Y	.	.	.	.	-	.	.	.	.	.	.	.	.	.	.	.	.	.	.	.	.	.	.	.
	Pakistan: Palas valley, PV2	.	.	.	.	.	.	.	-	.	.	.	.	.	.	.	.	.	.	.	.	.	.	.	.	.	.	.
	Pakistan: Hazara, HZ3	.	.	.	.	.	.	.	-	.	.	.	.	.	.	.	.	.	.	.	.	.	.	.	.	.	.	.
	India: Himanchal Pradesh, FU1	.	.	.	.	.	.	.	-	.	.	.	.	.	.	.	.	.	.	.	.	.	.	.	.	.	.	.
	India: Uttranchal, Chamoli, IN74	.	.	.	.	.	.	.	-	.	.	.	.	.	.	.	.	.	.	.	.	.	.	.	.	.	.	.
	Nepal: Darchula, DO16	.	.	.	.	.	.	.	-	.	.	.	.	.	.	.	.	.	.	.	.	.	.	.	.	.	.	.
	Nepal: Jumla, JD1	.	.	.	.	.	.	.	-	.	.	.	.	.	.	.	.	.	.	.	.	.	.	.	.	.	.	.
	Nepal: Manang, MC21	.	.	.	.	.	.	.	-	.	.	.	.	.	.	.	.	.	.	.	.	.	.	.	.	.	.	.
	Nepal, Baglung, BB1	.	.	.	.	.	.	.	-	.	.	.	.	.	.	.	.	.	.	.	.	.	.	.	.	.	.	.
	China: Tibet, Jilong, GL11	.	.	.	.	.	.	.	-	.	.	.	.	.	.	.	.	.	.	.	.	.	.	.	.	.	.	.
T. mairei	Nepal: Kavre, KD6	C	C	.	A	A	.	C	-	C	C	.	C	T	T	.	C	C	C	T	.	.	T	C	C	C	T	G
	Nepal: Sindhuli, SR5	C	C	.	A	A	.	C	-	C	C	.	C	T	T	.	C	C	C	T	.	.	T	C	C	C	T	G
	Nepal: Sindhuli, SM1	C	C	.	A	A	.	C	-	C	C	.	C	T	T	.	C	C	C	T	.	.	T	C	C	C	T	G
	India: Meghalaya, IN1	C	C	.	A	A	.	C	-	C	C	.	C	T	T	.	C	C	C	T	.	.	T	C	C	C	T	G
	Myanmar: Hilawng ridge, BU1	C	C	.	A	A	.	.	-	C	C	.	C	T	T	.	C	C	C	T	.	.	T	C	C	C	T	G
	Vietnam: Lam Dong, VN28	C	C	.	A	A	.	.	-	C	.	.	.	T	T	.	C	C	C	T	.	.	T	C	.	C	T	G
	China: Jiangxi, JX5	C	C	.	A	A	.	.	-	C	.	.	.	T	T	.	C	C	C	T	.	.	T	C	.	C	T	G
	China: Guizhou, LS20	C	C	.	A	A	.	C	-	C	.	.	.	T	T	.	C	C	C	T	.	.	T	C	.	C	T	G
T. wallichiana	Nepal: Baglung, BH10	.	.	.	.	A	G	.	T	.	.	A	.	T	T	T	.	C	C	.	C	.	.	C	.	C	T	G
	Nepal: Kaski, KC1	.	.	.	.	A	G	.	T	.	.	A	.	T	T	T	.	C	C	.	C	.	.	C	.	C	T	G
	Nepal: Gorkha, GK5	.	.	.	.	A	G	.	T	.	.	A	.	T	T	T	.	C	C	.	C	.	.	C	.	C	T	G
	Nepal: Rasuwa, RT8	.	.	.	.	A	G	.	T	.	.	A	.	T	T	T	.	C	C	.	C	.	.	C	.	C	T	G
	Nepal: Sagarmatha, ND16	.	.	.	.	A	G	.	T	.	.	A	.	T	T	T	.	C	C	.	C	.	.	C	.	C	T	G
	Nepal: Taplejung, TK1	.	.	.	.	A	G	.	T	.	.	A	.	T	T	T	.	C	C	.	C	.	.	C	.	C	T	G
	Bhutan: Thimpu, BT1	.	.	.	.	A	G	.	T	.	.	A	.	T	T	T	.	C	C	.	C	.	.	C	.	C	T	G
	China: Xizang, Yadong, XY24	.	.	.	.	A	G	.	T	.	.	A	.	T	T	T	.	C	C	.	C	.	.	C	.	C	T	G
	China: Xizang, Cuona, CN17	.	.	.	.	A	G	.	T	.	.	A	.	T	T	T	.	C	C	.	C	C	.	C	.	C	T	G
	China: Xizang, Chayu, CY01	.	.	.	.	A	G	.	T	.	.	A	.	T	T	T	.	C	C	.	C	.	.	C	.	C	T	G
	China: Yunnan, Gongshan,GS1	.	.	.	.	A	G	.	T	.	.	A	.	T	T	T	.	C	C	.	C	.	.	C	.	C	T	G
	China: Yunnan, Lushi, LK01	.	.	.	.	A	G	.	T	.	.	A	.	T	T	T	.	C	C	.	C	.	.	C	.	C	T	G
	China: Yunnan, Yingjiang, YJ01	.	.	.	.	A	G	.	T	.	.	A	.	T	T	T	.	C	C	.	C	.	.	C	.	C	T	G
**Hybrid individual**	Nepal: Sindhupalchok, SL18	.	.	.	.	W	S	.	Y	.	.	M	.	Y	Y	Y	.	M	M	.	Y	.	.	M	.	Y	K	K
**Hybrid individual**	Nepal: Sindhupalchok, SL19	.	.	.	.	W	S	.	Y	.	.	M	.	Y	Y	Y	.	M	M	.	Y	.	.	M	.	Y	K	K

Character states shown in comparison to accession *T. contorta*, KV10, identical states indicated by "." in other accessions, "-" = indel.
