# Supplementary material for: Using Morphological, Molecular and Climatic Data to Delimitate Yews along the Hindu Kush-Himalaya and Adjacent Regions
Source: PLoS One. 2012 Oct 8;7(10):e46873. doi: 10.1371/journal.pone.0046873 (PMC3466193; doi:10.1371/journal.pone.0046873)
Supplement: Table S5 — trn L-F sequence matrix. Variable position of the cpDNA sequences (trnL-F) of 36 accessions sampled across the distribution range of all three species of Taxus. (PDF) [file pone.0046873.s011.pdf]

Supplementary Table S5

Table S5. *trn*L-F sequence matrix. Variable position of the cpDNA sequences (*trn*L-F) of 36 accessions sampled across the distribution range of all three species of *Taxus*.

**Species**	**Country/Locality/Code**	**Position**														
		**102**	**238**	**297**	**314**	**328**	**338**	**345**	**448**	**513**	**542**	**690**	**688**	**756**	**770**	**775**
T. contorta	Pakistan: Kurram Valley, KV10	C	A	G	T	T	C	G	G	0	A	C	G	0	C	G
	Pakistan: Tirah, TH15	.	.	.	.	.	.	.	.	0	.	.	.	0	.	.
	Pakistan: Chitral, CH5	.	.	.	.	.	.	.	.	0	.	.	.	0	.	.
	Pakistan: Palas valley, PV2	.	.	.	.	.	.	.	.	0	T	.	.	0	.	.
	Pakistan: Hazara, HZ3	.	.	.	.	.	.	T	.	0	.	.	.	0	.	.
	India: Himanchal Pradesh, FU1	.	.	.	.	.	.	.	.	0	.	.	.	0	.	.
	India: Uttranchal, Chamoli, IN74	.	.	.	.	.	.	.	.	0	.	.	.	0	.	.
	Nepal: Darchula, DO16	.	.	.	.	.	.	.	.	0	.	.	.	0	.	.
	Nepal: Jumla, JD1	.	.	.	.	.	.	.	.	0	.	.	.	0	.	.
	Nepal: Manang, MC21	.	.	.	.	.	.	.	.	0	.	.	.	0	A	.
	Nepal, Baglung, BB1	.	.	.	.	.	.	.	.	0	.	.	.	0	.	.
	China: Tibet, Jilong, GL11	A	.	.	.	.	.	.	.	0	.	.	.	0	.	.
T. mairei	Nepal: Kavre, KD6	.	.	T	.	G	.	.	.	0	.	A	.	1^{d}	.	.
	Nepal: Sindhuli, SR5	.	.	T	.	G	.	.	.	0	.	A	.	1^{d}	.	.
	Nepal: Sindhuli, SM1	.	.	T	.	G	.	.	.	0	.	A	.	1^{d}	.	.
	Bhutan: Punakha-Tashitang, BT5	.	.	T	.	G	.	.	.	0	.	A	.	1^{d}	.	.
	India: Meghalaya, IN1	.	.	T	.	G	.	.	.	0	.	A	.	1^{d}	.	.
	Myanmar: Hilawng ridge, BU1		.	T	.	G	.	.	.	0	.	A	.	1^{d}	.	.
	Vietnam: Lam Dong, VN28	.	.	T	.	G	.	.	.	0	.	A	.	1^{d}	.	.
	China: Jiangxi, JX5	.	.	T	.	G	.	.	.	0	.	A	.	1^{d}	.	.
	China: Guizhou, LS20	.	.	T	.	G	.	.	.	0	.	A	.	1^{d}	.	.
T. wallichiana	Nepal: Baglung, BH10	.	.	T	.	G	T	.	.	1^{b}	.	A	T	1^{e}	A	T
	Nepal: Kaski, KC1	.	.	T	G	G	T	.	T	1^{a}	.	A	T	1^{e}	A	T
	Nepal: Gorkha, GK5	.	.	T	.	G	T	.	T	1^{a}	.	A	T	1^{e}	A	T
	Nepal: Rasuwa, RT8	.	C	T	.	T	T	.	T	1^{a}	.	A	T	1^{e}	A	T
	Nepal: Sagarmatha, ND16	.	.	T	.	G	T	.	T	1^{a}	.	A	T	1^{e}	A	T
	Nepal: Taplejung, TK1	.	.	T	.	G	T	.	T	1^{a}	.	A	T	1^{e}	A	T
	Bhutan: Thimpu, BT1	.	.	T	.	G	T	.	T	1^{a}	.	A	T	1^{e}	A	T
	China: Xizang, Yadong, XY24	.	.	T	.	G	T	.	.	0	.	A	T	1^{e}	A	T
	China: Xizang, Cuona, CN17	.	.	T	.	G	T	.	.	0	.	A	T	1^{e}	A	T
	China: Xizang, Chayu, CY01	.	.	T	.	G	T	.	.	1^{a}	.	A	T	1^{e}	A	T
	China: Yunnan, Gongshan,GS1	.	.	T	.	G	T	.	.	1^{c}	.	A	T	1^{e}	A	T
	China: Yunnan, Lushi, LK01	.	.	T	.	G	T	.	T	1^{a}	.	A	T	1^{e}	A	T
	China: Yunnan, Yingjiang, YJ01	.	.	T	.	G	T	.	T	1^{a}	.	A	T	1^{e}	A	T
**Hybrid individual**	Nepal: Sindhupalchok, SL18	.	.	.	.	.	.	.	.	0	.	.	.	0	.	.
**Hybrid individual**	Nepal: Sindhupalchok, SL19	.	.	.	.	.	.	.	.	0	.	.	.	0	.	.

Character states shown in comparison to accession *T. contorta*, KV10, identical states indicated by "." in other accessions. 0= absent; 1^{a}= AGAAAGATCAAATATTATTA; 1^{b}= AGAAAGATCAAATATTATTAAGAAAGATCAAATATTATTA; 1^{c}= AGAAATATCAAATATTATTA; 1^{d} =AAAAAA-G; 1^{e} = AAAAAAAG
