# Supplementary material for: Using Morphological, Molecular and Climatic Data to Delimitate Yews along the Hindu Kush-Himalaya and Adjacent Regions
Source: PLoS One. 2012 Oct 8;7(10):e46873. doi: 10.1371/journal.pone.0046873 (PMC3466193; doi:10.1371/journal.pone.0046873)
Supplement: Table S6 — Statistics of univariate ANOVA among Taxus of the Hindu Kush-Himalaya and adjacent regions for 19 bioclimatic variables. (PDF) [file pone.0046873.s012.pdf]

Supplementary Table S6

Table S6. Statistics of univariate ANOVA among *Taxus* of the Hindu Kush-Himalaya and adjacent regions for 19 bioclimatic variables.

Variables		Sum of Squares	df	Mean Square	F	Sig.	Tukey *Post-hoc* test
Bio1	Between species	792.32	2	396.16	70.48	0.000	T.contorta/T. wallichiana (*p*=0.524)
Bio2	Between species	50.25	2	25.13	21.42	0.000	T.contorta/T. mairei (*p*=0.143)
Bio3	Between species	2452.35	2	1226.18	39.24	0.000	*p*=0.000
Bio4	Between species	1327968.66	2	663984.33	65.65	0.000	*p*=0.000
Bio5	Between species	306.76	2	153.38	16.62	0.000	*p*=0.000
Bio6	Between species	1530.46	2	765.23	88.29	0.000	T.contorta/T. wallichiana (*p*=0.999)
Bio7	Between species	733.89	2	366.95	47.33	0.000	*p*=0.000
Bio8	Between species	590.79	2	295.39	35.16	0.000	*p*=0.000
Bio9	Between species	1237.42	2	618.71	77.21	0.000	*p*=0.000
Bio10	Between species	235.11	2	117.55	16.29	0.000	T.contorta/T. wallichiana (*p*=0.358)
Bio11	Between species	1709.99	2	855.00	120.42	0.000	*p*=0.000
Bio12	Between species	32690000.00	2	16350000.00	44.37	0.000	*p*=0.000
Bio13	Between species	1718425.31	2	859212.66	31.04	0.000	*p*=0.000
Bio14	Between species	2285.35	2	1142.68	29.74	0.000	T.mairei/T. wallichiana (*p*=0.996)
Bio15	Between species	17492.01	2	8746.00	34.98	0.000	T.mairei/T. wallichiana (*p*=0.291)
Bio16	Between species	12570000.00	2	6285132.67	35.76	0.000	*p*=0.000
Bio17	Between species	91758.03	2	45879.02	55.71	0.000	T.mairei/T. wallichiana (*p*=0.8)
Bio18	Between species	9863941.93	2	4931970.96	28.04	0.000	*p*=0.000
Bio19	Between species	479754.92	2	239877.46	75.14	0.000	T.mairei/T. wallichiana (*p*=0.989)
